# A Novel Image-Based Screening Method to Study Water-Deficit Response and Recovery of Barley Populations Using Canopy Dynamics Phenotyping and Simple Metabolite Profiling

**DOI:** 10.3389/fpls.2019.01252

**Published:** 2019-10-15

**Authors:** Cintia F. Marchetti, Lydia Ugena, Jan F. Humplík, Michal Polák, Sanja Ćavar Zeljković, Kateřina Podlešáková, Tomáš Fürst, Nuria De Diego, Lukáš Spíchal

**Affiliations:** ^1^Department of Molecular Biology, Centre of the Region of Haná for Biotechnological and Agricultural Research, Faculty of Science, Palacký University, Olomouc, Czechia; ^2^Department of Chemical Biology and Genetics, Centre of the Region Haná for Biotechnological and Agricultural Research, Faculty of Science, Palacký University, Olomouc, Czechia; ^3^Department of Phytochemistry, Centre of the Region Haná for Biotechnological and Agricultural Research, Faculty of Science, Palacký University, Olomouc, Czechia; ^4^Department of Genetic Resources for Vegetables, Medicinal and Special Plants, Centre of the Region Haná for Biotechnological and Agricultural Research, Crop Research Institute, Olomouc, Czechia; ^5^Department of Mathematical Analysis and Applications of Mathematics, Faculty of Science, Palacky University, Olomouc, Czechia

**Keywords:** amino acids, antioxidative enzymes, canopy height, fluorescence, *Hordeum vulgare*, indoor phenotyping, polyamines, red, green, blue (RGB) imaging

## Abstract

Plant phenotyping platforms offer automated, fast scoring of traits that simplify the selection of varieties that are more competitive under stress conditions. However, indoor phenotyping methods are frequently based on the analysis of plant growth in individual pots. We present a reproducible indoor phenotyping method for screening young barley populations under water stress conditions and after subsequent rewatering. The method is based on a simple read-out of data using RGB imaging, projected canopy height, as a useful feature for indirectly following the kinetics of growth and water loss in a population of barley. A total of 47 variables including 15 traits and 32 biochemical metabolites measured (morphometric parameters, chlorophyll fluorescence imaging, quantification of stress-related metabolites; amino acids and polyamines, and enzymatic activities) were used to validate the method. The study allowed the identification of metabolites related to water stress response and recovery. Specifically, we found that cadaverine (Cad), 1,3-aminopropane (DAP), tryptamine (Tryp), and tyramine (Tyra) were the major contributors to the water stress response, whereas Cad, DAP, and Tyra, but not Tryp, remained at higher levels in the stressed plants even after rewatering. In this work, we designed, optimized and validated a non-invasive image-based method for automated screening of potential water stress tolerance genotypes in barley populations. We demonstrated the applicability of the method using transgenic barley lines with different sensitivity to drought stress showing that combining canopy height and the metabolite profile we can discriminate tolerant from sensitive genotypes. We showed that the projected canopy height a sensitive trait that truly reflects other invasively studied morphological, physiological, and metabolic traits and that our presented methodological setup can be easily applicable for large-scale screenings in low-cost systems equipped with a simple RGB camera. We believe that our approach will contribute to accelerate the study and understanding of the plant water stress response and recovery capacity in crops, such as barley.

## Introduction

Abiotic stresses, in particular water deficit, constrain the global production of crops, affecting both the vegetative and reproductive phases of development (Wang et al., 2003). Not all water stress scenarios are equivalent; however, because the severity, frequency, duration, and timing of the stress can vary, and with them the impact on the plant (Chen et al., 2014). A robust and reliable analysis of phenotypic traits is, therefore, essential for each context to shed light on the basic tolerance mechanisms and develop strategies for breeding crops that are more tolerant to adverse environments.

Barley is the fourth most cultivated crop worldwide (http://www.fao.org/faostat/en/#data/QC). As a diploid organism, it is considered to be a suitable model for studying the more complex polyploid species belonging to the *Triticeae* tribe. Under water stress conditions, barley displays reduced growth and adaptations of other physiological parameters, such as chlorophyll (Chl) content, net photosynthetic rate, and water content (Ahmed et al., 2013; Saade et al., 2018). However, the mechanisms that confer water stress tolerance and recovery capacity in this crop species are still not fully understood.

Plant phenotyping platforms provide the potential for automated, fast scoring of several traits related to stress tolerance over a time-course, using non-invasive sensors (Granier et al., 2006; Chen et al., 2014; Humplík et al., 2015; Awlia et al., 2016; De Diego et al., 2017). Until now, published indoor protocols have been based on the response of individual plants often restricted by pot growth, whereas in the field, plant growth results in a canopy (Araus and Cairns, 2014). Nevertheless, in the field, plant growth is seasonally dependent, often reducing the number of possible experiments to one per year, whereas indoor phenotyping allows continuous repetition of experiments. Furthermore, screening for specific tolerance traits under controlled conditions is often necessary to manage the complexity of interactions between genotype and environment on the phenotype (Ghanem et al., 2015). The specific metric being monitored, therefore, needs to be defined, and its relationship with the physiological trait of interest has to be resolved and validated. The plant phenotype is also determined by complex genome–environment–management interactions: the sum of the complex interactions between metabolic pathways and intracellular regulatory networks are reflected in internal, physiological, and biochemical phenotypes (Großkinsky et al., 2015). Thus, a characterization limited only to a detailed description of a set of image-based traits remains incomplete for understanding plant responses to drought. An integration of data from phenomics with other “-omics” (e.g., metabolomics and genomics) may help dissect the plant response and clarify the key traits involved in the mechanisms of stress tolerance and acclimation. As an example, the combination of phenomics with metabolomics can help to identify metabolites that are mainly accumulated under water stress conditions. Particularly, some metabolites, such as polyamines (PAs) and some amino acids (AAs) involved in glutamate metabolism like γ-amino butyric acid (GABA) and l-proline (Pro), play an essential role regulating stress tolerance like compatible solutes contributing to osmotic potential, mediators of antioxidant responses or signal molecules (De Diego et al., 2013; Podlešáková et al., 2019). Concretely, GABA, Pro, and l-arginine (Arg) have been described as metabolites involved in plant recovery and hardening under drought stress conditions (De Diego et al., 2015).

We present a non-destructive method for studying the water stress tolerance and recovery on a population of barley based on image analysis of canopy height using a simple red, green, blue (RGB) camera. To introduce the method, we used barley cultivar Golden Promise as a good representative of the varieties with agronomical interest due to its semi-dwarf with low lodging problems (Thomas et al., 1984) and stress tolerance (Forster et al., 2000). Besides, this cultivar is highly transformable (Harwood, 2012), so it is very interesting for studying the stress response in barley. Moreover, we focused on profiling the primary and secondary metabolites, AAs, and PAs, which play a dual role as tolerance indicators and signal molecules (Muscolo et al., 2015; Urano et al., 2010) to study further the involvement of these molecules regulating plant water stress response and recovery capacity. We show that, in combination with other -omic approaches, such as metabolomics, followed by multivariate statistical methods, the experimental procedures for studying stress response can be made more efficient. The result is a simple and highly reproducible method for studying the stress response and recovery in barley populations, which is applicable for breeding programs to select and characterize potentially successful varieties.

## Materials and Methods

### Plant Material and Growth Conditions

**Figure 1:**
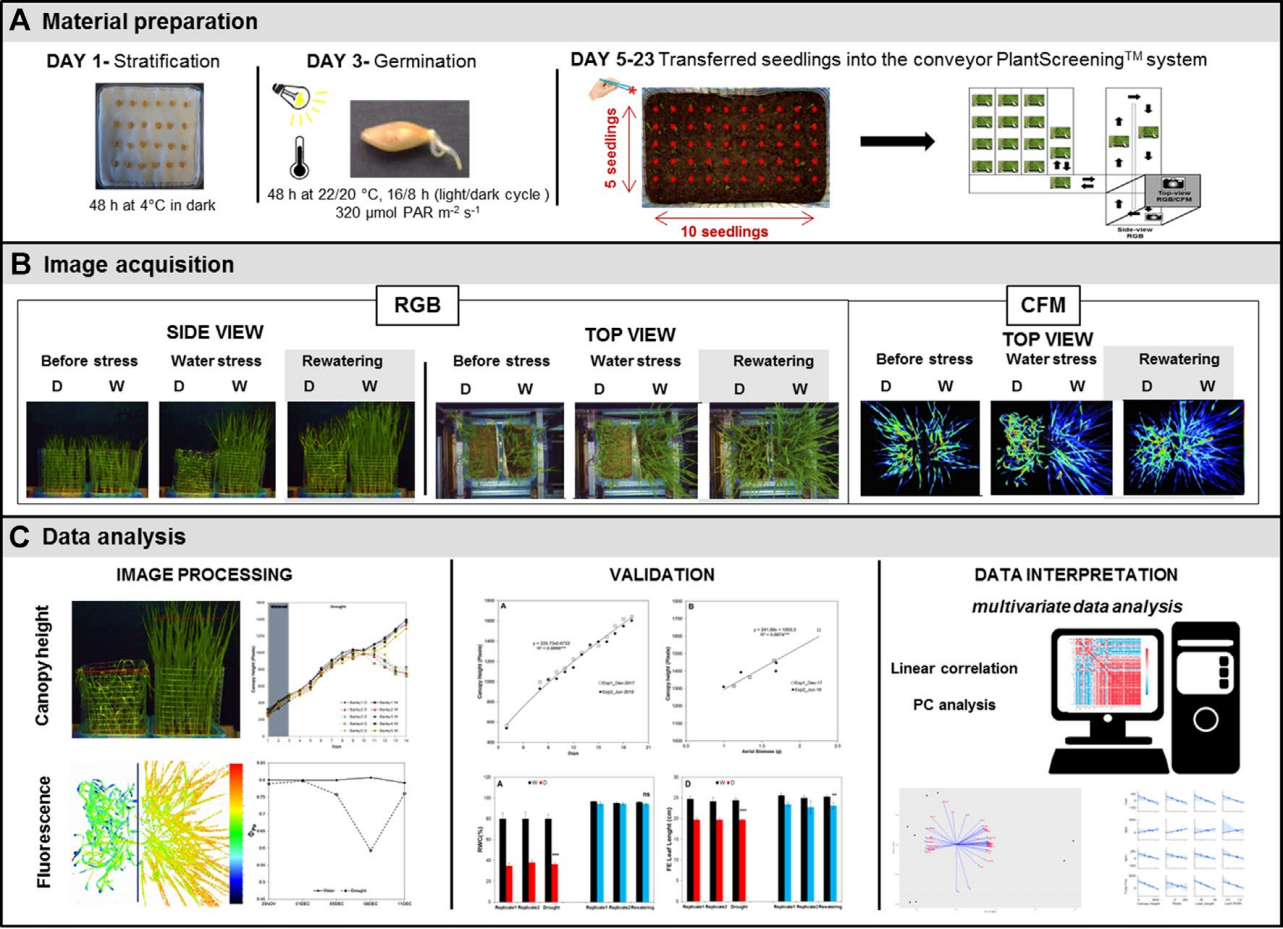
Scheme of the protocol used for non-invasive phenotyping of barley (*Hordeum vulgare*) seedlings growing under water stress conditions. **(A)** Barley seeds were germinated on filter paper and 50 seedlings of similar radicle size were transplanted into soil in standardized PlantScreen™ measuring trays. **(B)** The trays were transferred to an XYZ PlantScreen™ chamber with a conveyor system for automatic image acquisition. **(C)** The canopy height was analyzed using an in-house software routine implemented in MatLab R2015, and the data were evaluated by multivariate statistical analyses using Phyton version 3.6.5 and R version 3.5.1.

Barley (*Hordeum vulgare*) cultivar Golden Promise was used for the study. Seeds were surface-sterilized by soaking in 70% ethanol for 30 s, and then washed three times with sterilized water, and immediately after that with a 4% solution of sodium hypochlorite for 4 min followed by an additional four washes with sterilized water. The sterile seeds were placed on wet tissue paper inside square plastic plates (120 × 120 mm) and maintained for 2 days at 4°C in the dark for imbibition, as schematized in **Figure 1**. The plates were then transferred into a growth-chamber, under controlled conditions of a 22°C, 16:8-h light/dark cycle with a photosynthetically active radiation (PAR) light intensity of 120 µmol photons m^−2^ s^−1^ for 2 days, to induce germination (**Figure 1**).

To demonstrate the applicability of the method, three different transgenic lines with silenced gene A (transgenic lines 1, 2, and 3) were characterized and compared with wt grown under water stress conditions and after rewatering. A construct generating a hairpin RNA structure (hpRNA) was prepared to silence the candidate gene. Two copies of the same 196-bp fragment from the gene A were inserted in the antisense and sense orientation in the vector pBract207 (provided by John Innes Centre, Norwich, United Kingdom), downstream of the maize ubiquitin-1 promoter. The final silencing cassette contained the hygromycin (hyg) phosphotransferase gene (conferring hyg resistance) driven by the cauliflower mosaic virus 35S promoter at the left border, and the terminator of the nopaline synthase gene adjacent to the right border. The final vector was sequenced (SeqMe, Czechia) to confirm the right orientation of both copies of the targeted gene and then electrotransformed into *Agrobacterium tumefaciens* strain AGL1 together with the helper vector pSoup. Transgenic barley plants were prepared according to Harwood (2012) using *A. tum*.-mediated transformation of immature embryos. All obtained transgenic lines were screened for the presence of sense and antisense components of the silencing cassette in successive generations. Moreover, the DNA ploidy levels of the obtained transformants were determined by flow cytometry, and the number of T-DNA insertions was assessed by DNA Southern hybridization, as described in Holubová et al. (2018).

### Non-Invasive Plant Phenotyping

#### Phenotyping Platform, Experimental Setup, and Assay Conditions

To develop the barley screening method, first, we performed and analyzed reproducibility of two independent assays using well-watered conditions (1^st^ experiment—December 2017 and 2^nd^ experiment—June 2018; **Supplementary Table 1**). After that, we performed a final experiment to evaluate the growth of barley seedling population under water deficit (3^rd^ experiment—**Supplementary Table 1**). In all experiments, the barley seedlings were planted in plastic containers (32 × 19.5 × 10 cm, with a volume of 4 l) filled with 2.8 kg of a 2:1 mixture of substrate (Substrate 2; Klasmann-Deilmann GmbH, Geeste, Germany) and sand. A total of 50 seedlings per container was sown, distributed in five rows containing 10 plants each, with a final density of 1000 plants m^–2^ (**Figure 1**). Two containers with 50 seedlings each were then placed together in a standard PlantScreen^™^ measuring tray (Photon Systems Instruments, Brno, Czech Republic).

For the image analysis, the tray with two containers housing 50 plants each was transferred onto an OloPhen platform (http://www.plant-phenotyping.org/db_infrastructure#/tool/57). In the water deficit experiment a metal mesh was installed for better separation of the leaves from the variants [well-watered (W) and water stress-drought (D)] in each container (**Figure 1**). The trays were located into the PlantScreen^™^ conveyor system installed in a growth chamber with a controlled environment and LED lighting (Photon Systems Instruments). The growth conditions were set to simulate a long day, with a regime of 22°C to 20°C in a 16:8-h light/dark cycle, a PAR irradiance of 320 μmol photons m^−2^ s^−1^ and a relative humidity of 40%.

#### Imaging Acquisition

The PlantScreen^™^ system is equipped with a top-view and side-view RGB camera and top-view FluorCam (**Figure 1**). The RGB and FluorCam images were automatically stored in a database server. The images were then evaluated. The side-view RGB images were analyzed using an in-house software routine implemented in MatLab R2015 that was developed and validated by the authors of this study. The application can be used without charge by obtaining a license from Palacký University, by emailing Tomáš Fürst (tomas.furst@upol.cz) and agreeing not to use the application for commercial purposes.

To provide a comparative view, we analyzed Chl fluorescence-related parameters throughout the experiments using a top-view FluorCam. A standard protocol was used for the measurement of Chl fluorescence quenching using the Chl fluorescence imaging (CFIM) part of the PlantScreen™ platform using the same protocol described by Humplík et al. (2015). Image data were processed, and Chl fluorescence parameters were calculated using software FluorCam 7 (Photon Systems Instruments). Thus, we estimated: (a) the maximum quantum yield of photosystem II (PSII) photochemistry for a dark-adapted state, Φ_Po_; (b) the actual quantum yield of PSII photochemistry for a light-adapted state, Φ_P_; (c) the quantum yield of constitutive non–light-induced (basal or dark) dissipation processes consisting of Chl fluorescence emission and heat dissipation, Φ_(f,D)_, quantum yield of regulatory light-induced non-photochemical quenching, Φ_NPQ_. It is worth mentioning that Φ_P_ + Φ_(f,D)_ + Φ_NPQ_ = 1, and Φ_P_ = *q*P Φ_PSII_, where *q*P = ((F_M_′ − F(t))/(F_M_′ − F_0_′)) is the coefficient of photochemical quenching, which estimates a fraction of the so-called open PSII reaction centers, and that Φ_PSII_ = ((F_M_′ − F_0_′)/F_M_′) is the maximal quantum yield of the PSII photochemistry for a light-adapted state.

#### Watering Regime

In each container, the substrate water content (%) was calculated as follows; the substrate was weighted and then watered to full capacity, and the top was covered with plastic bags for reducing evaporation losses. When the water stopped draining from the container (c. 10 h later) the weight was again measured. The substrate was then dried for 48 h at 105°C until complete dryness, and the weight was also determined. The three weights of the substrate water moisture status were used for calculating the gravimetric water content (θg) and substrate maximum water holding capacity. All containers were watered with tap water (average conductivity *c.* 56 mS m^−1^) at 100% of field capacity after sowing. Then, the control plants were irrigated every second day to maintain 80% of field capacity until the end of the experiment. In the 3rd experiment, the irrigation was stopped in the water stress variant when the plants presented the first fully expanded leaf. When the substrate water content decreased to 65% (day 15), the water limited variants were rewatered at full water capacity (100%).

### The Assay Workflow

To study water deficit and recovery in barley populations using imaging acquisition approaches, we established the protocol schematized in **Figure 1**. The protocol takes a total of 3 weeks, including seed germination (2 days), seedling growth under water stress conditions (16 days) and subsequent rewatering (4 days). After imbibition, a total of 50 germinated seeds were transferred to plastic containers (**Figure 1**). The selection of germinated seeds at a similar developmental stage (radicle length) is important to reduce within-population variability. Two randomly selected containers were then placed in standardized trays and transported within the PlantScreen™ on conveyor belts. Automated RGB imaging from two optical projections (top- and side-view) was performed every day for the next 19 days. The daily RGB measurements of 16 trays (1600 seedlings) took 20 min in total. When the top-view fluorescence intensity measurement was included (every third day), the total process time increased to 4 h. The PlantScreen^™^ Analyzer software processed the raw data automatically. Raw data were automatically stored in PlantScreen database from where they were subsequently exported for further image processing and analysis.

Side-view imaging allowed separation of the plants from the background and the differentiation between the left and right containers using a pre-defined vertical line in the image based on the metal mesh installed between them (**Figure 1**). To define and evaluate the canopy height in each container (or variant) automatically, the green mask of the plant was found using a thresholding algorithm. A line was then placed above the upper most pixel of the mask. The line was gradually lowered (each step by one pixel) and the following criterion evaluated for each possible position of the line:

crit=A/B

where A is the number of pixels on a line that also belong to the mask, and B is the length of the line (in pixels). Thus, the criterion represents the fraction of the line that intersects the mask. Once this fraction exceeded a user-defined threshold, the process was stopped, and the position of the line was recorded. This position was then used as a proxy for canopy height.

### Manual Parameters

#### Biometric Parameters

The aerial biomass of the seedlings was determined from the fresh weight (FW, g) by cutting the shoots 1 cm above ground level from eight or five plants per replicate and variant at the end of the water stress period and after 4 days of rewatering, respectively. The developmental stage of each plant at the same time points, according to the Biologische Bundesanstalt, Bundessortenamt and CHemical industry (BBCH) scale (Earth Observation and Research Branch Team, 2011), and the size (length and width) of the youngest and fully expanded leaf were also evaluated.

#### Plant Water Status

The relative water content (RWC, %) was measured in five individual plants per variant and replicate. The measurement was performed using 2 cm from the middle part of the last youngest mature leaf collected at the end of the water stress period and after rewatering. RWC was calculated using the following equation: RWC (%) = (FW − DW)/(TW − DW) × 100, where FW is the fresh weight at harvesting time, TW is the total weight as total turgor estimated after 24 h of imbibition, and DW is the dry weight after 48 h at 85°C.

#### Chl Content

The index of relative Chl content or “greenness” of leaves was measured *in vivo* using a portable SPAD-502 Plus Chl meter (Konica Minolta Inc.). Six measurements were taken in the last youngest mature leaf in five individual plants per replicate and variant at the end of the water stress period and after rewatering.

#### Antioxidant Enzymes

For the enzymatic analysis, three groups of five plants per replicate and variant after water stress and rewatering were collected and immediately frozen in liquid nitrogen. Before extraction, the five plants per each group were grounded by mortar and pestle using liquid nitrogen. The resulting pool from each replicate and variant was divided into three analytical replicates for quantification of antioxidant activity. One hundred milligrams of grounded material was homogenized at a ratio of 1:5 with extraction buffer [50 mM Tris (pH 7.6), containing 2 mM magnesium sulphate, 1 mM ethylenediaminetetraacetic acid (EDTA), 1 mM ascorbic acid, 1 mM phenylmethylsulfonyl fluoride and 0.5% (v/v) Triton X100] in the presence of polyvinylpolypyrrolidone. After centrifugation at 19,000*g* for 20 min at 4°C, the supernatant was collected and centrifuged under the same conditions for an additional 10 min. The concentration of proteins in the crude extract was evaluated using the method described by Bradford (1976). Catalase (CAT) activity (µmol min^–1^ mg^–1^ total protein) was measured according to Aebi (1984). Briefly, the decrease in absorbance at 240 nm of a reaction mixture consisting of 25 μl enzymatic extract in a final volume of 1ml reaction mixture containing 50 mM potassium phosphate (pH 7.0) and 25 mM H_2_O_2_ was measured. The molar extinction coefficient of 38 M^–1^ cm^–1^ was used to calculate CAT activity.

Ascorbate peroxidase (APX) and guaiacol peroxidase (POX) activity was determined according to Prochazkova et al. (2001). APX activity was deduced from the decrease in ascorbate concentration, seen as a decline in the optical density at 290 nm. A value for the activity was calculated using an extinction coefficient of 2.8 mM^–1^ cm^–1^ for the ascorbate during 30 s. The assay was performed in a final volume of 1 ml containing 50 mM potassium phosphate (pH 7.0), 1.7mM H_2_O_2_, 0.3mM ascorbic acid, and 85μl enzymatic extract. POX activity was determined from the increase in formation of tetraguaiacol, seen as an increase in the optical density at 470 nm. The activity was calculated using an extinction coefficient 26.6 mM^–1^ cm^–1^ during 30 s. The assay was carried out using a reaction mixture consisting of 4 μl extract, 150 mM potassium phosphate (pH 6.1), 8 mM guaiacol and 2.2 mM H_2_O_2_.

#### Metabolite Quantification

For the free AA analysis, three groups of five individual plants per replicate and variant were collected after water stress and rewatering. All individuals from each group were pooled together using liquid nitrogen. Five milligrams FW of the pooled material was measured and extracted. The extraction procedure was performed using a AccQTag Ultra derivatization kit (^©^Waters). All extracted samples were analyzed using an ACQUITY UPLC^®^ System and a XevoTM 122 TQ-S triple quadrupole mass spectrometer (^©^Waters) according to the annotation note of Waters Corporation (Milford, MA, USA) (Gray and Plumb, 2016). Calibration curves were constructed for each component analyzed using internal standards: γ-aminobutyric acid (GABA), l-alanine (Ala), l-arginine (Arg), l-asparagine (Asn), l-aspartic acid (Asp), l-citrulline (Cit), l-glutamine (Gln), l-glutamic acid (Glu), l-glycine (Gly), l-histidine (His), l-ileucine (Ile), l-leucine (Leu), l-methionine (Met), l-phenylalanine (Phe), l-proline (Pro), l-serine (Ser), l-tryptophan (Trp), and l-tyrosine (Tyr), and the deuterium-labeled compounds l-glutamic acid-2,3,3,4,4-d_5_, γ-aminobutyric acid-2,2,3,3,4,4-d_6_ and dl-leucine-2,3,3,4,5,5,5,5′,5′,5′-d_10,_ all purchased from ^©^Sigma-Aldrich Inc. (Germany).

Free PAs [agmatine (Agm) as precursor, cadaverine (Cad), 1,3-diaminopropane (DAP), putrescine (Put), spermidine (Spd), spermine (Spm) tyramine (Tyra), and tryptamine (Tryp)] were isolated and derivatized by slightly modified method of Taibi et al. (2000). Diaminohexane (DAH) was used as internal standard. All the chemicals were purchased from Sigma Aldrich chemical company (St. Louis, MO, USA). Briefly, samples were dissolved in 50 µL of mobile phase (20% methanol in 15mM formic acid solution, pH 3.0) and analyzed immediately. UHPLC-MS/MS was performed on Nexera X2 UHPLC (Shimadzu Handels GmbH) coupled with a MS-8050 (Shimadzu Handels GmbH). Chromatographic separation was performed on an Acquity UPLC BEH C18 (100 × 2.1 mm; 1.7 µm particle size) column (Waters, Milford, MA, USA) with appropriate pre-column kept at 40°C. The mobile phase consisted of the mixture of aqueous solutions of 15mM formic acid, pH 3.0 (Solvent A) and methanol (solvent B). The analytes were separated *via* a binary gradient starting at 20% of B for 1 min, then increased to 50% for 0.1 min, isocratic at 50% for next 1.9 min, increased to 60% B for next 0.1 min, isocratic at 60% B for next 3.4 min, increased to 100% B for next 0.3 min, isocratic at 100% B for next 1.0 min, and decreased to 20% B for next 0.2 min. The equilibration to the initial conditions took an additional 4.0 min. The flow rate was 0.4 ml/min, and the injection volume was 5 µl. Benzoylated polyamines were detected in positive ionization mode ESI+ using appropriate MRM transitions. The mass spectra were obtained *via* electrospray ionization in positive mode with the following operating parameters: capillary voltage, −3 kV; interface voltage, 4 kV; interface temperature, 300°C; heating and drying gas flow, 10 l min_–1_; nebulizing gas flow, 3 l min_–1_. The concentration of AAs and PAs per dry weight (DW) was then calculated using the absolute water content of the plants.

### Statistical Analysis and Data Representation

To assess the differences between the variants of each non-invasive trait extracted from the image analysis, the non-parametric Kruskal Wallis one-way analysis of variance (ANOVA) was performed. For the validation of the method and to evaluate the influence of a tray position and the effect of the treatment (well-watered or water limited variant) in the morphological and physiological measurements at a particular timepoint, the two-way ANOVA was used. Different letters mean significant differences between variants. One-way ANOVA was used to analyze significant differences between the metabolites differed between the variants. Represented values annotated with ns indicate non-significant differences, and means annotated with asterisks indicate significant differences (*P ≤ 0.05; **P ≤ 0.01; ***P ≤ 0.001). Tukey HSD test was used for multiple comparison after ANOVA. All analyses were performed in R 3.5.1 software using *multcomp* package.

For multidimensional analyses, a principal component analysis (PCA) was carried out using the packages *factoextra, corrplot, PerformanceAnalytics*, and *ggpubr*, and the results were displayed in a biplot. The Pearson’s linear correlation coefficients between all pairs of studied variables and the significance were also represented in the correlation matrix and scatter plots.

## Results

### Phenotyping Method- Screening of Barley Population Using Simple RGB Imaging

#### (A) Experiment 1 and 2- Analysis of Projected Canopy Height in Barley Population at Control Conditions

**Figure 2:**
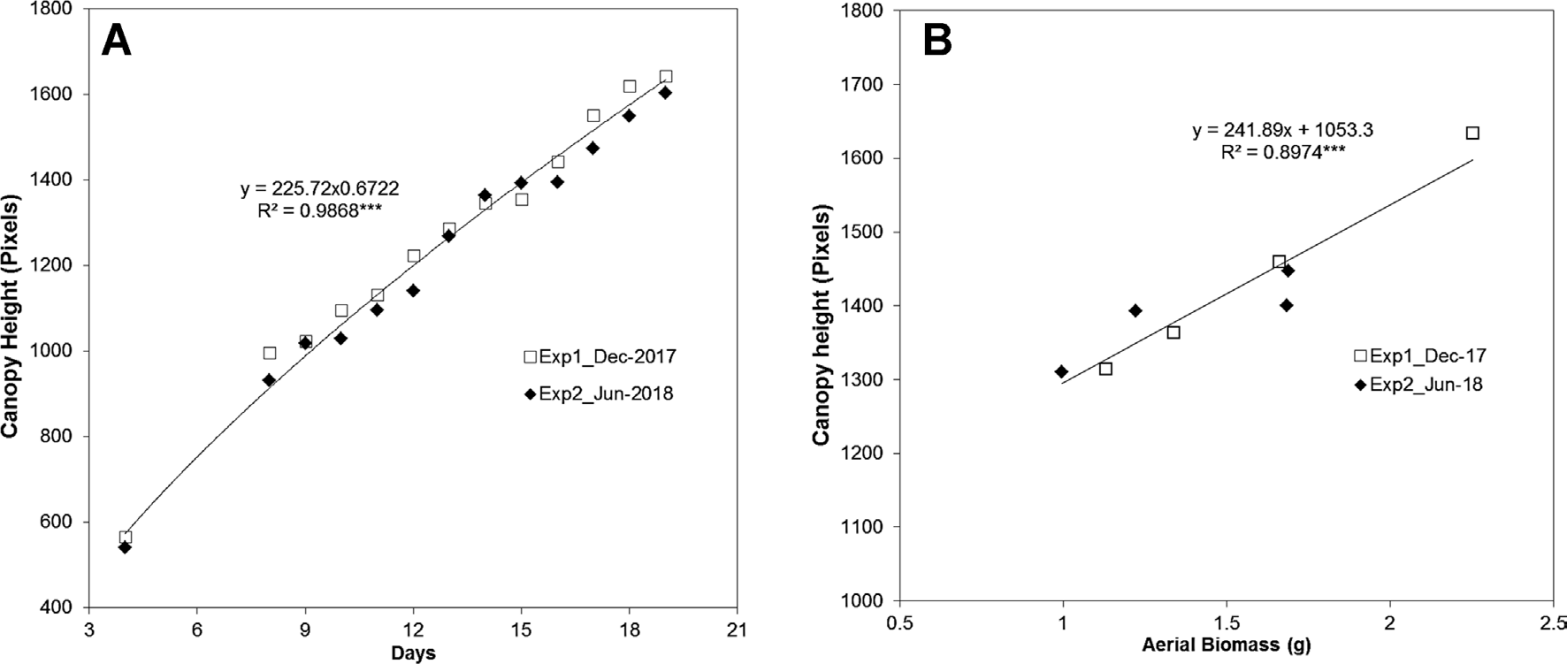
Analysis of the reproducibility of canopy height estimation in barley (*Hordeum vulgare*) seedlings from two independent experiments. **(A)** The reproducibility of projected canopy height dynamics (in pixels) in barley seedlings (n = 100) grown under control conditions in December 2017 and July 2018. **(B)** The correlation between canopy height and fresh aerial biomass (g) (n = 5) determined for replicates measured at day 16 and day 19 in barley seedlings from the two independent experiments. The regression curve and significance calculated from three independent trays is shown. ***P ≤ 0.001.

Applying the aforementioned approach, the side-view RGB images of the barley seedling canopy from two independent experiments (**Experiment 1** in December 2017 and **Experiment 2** in June 2018) were analyzed (**Supplementary Table S1**). Two randomly distributed trays containing 50 plants each were used per experiment. The reproducibility of the bioassay was corroborated, as the canopy height from both experiments presented the same trend line (**Figure 2A**).

A manual assessment of plants was then performed to complement and validate the traits derived from the automated plant imaging and image analysis, including the functionality of the hardware and software components. The aerial biomass in fresh weight (FW, g) from five seedlings randomly selected from the two different trays of each experiment was determined manually at days 16 and 19. A Pearson correlation-based comparison between the canopy height (pixels, from 50 plants) and the aerial biomass (the average of the five measured plants) revealed a highly significant correlation, with an *R* ^2^ of 0.90 (*P* < 0.001) (**Figure 2B**). This result provided clear evidence of the reliability of the method for analyzing shoot growth in the population of barley cv. Golden Promise.

#### (B) Experiment 3—Analysis of Projected Canopy Height in Barley Population Under Water Deficit and Subsequent Rewatering

**Figure 3:**
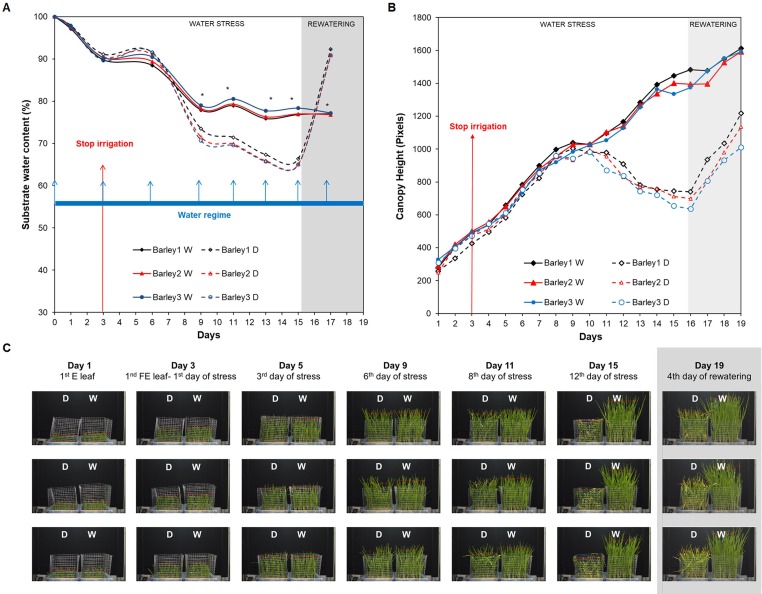
Dynamics of soil moisture and projected canopy height in barley seedlings growing under water stress conditions with subsequent rewatering. **(A)** Changes in substrate water content (%) of non-stressed (W, continuous lines) and stressed (D, discontinuous lines) barley seedlings (n = 50) from three independent trays (Barley 1, 2, and 3) grown for 13 days under water deficit conditions (with the endpoint at day 16) and subsequently rewatered for 4 days (with the endpoint at day 19). The watering regime consisted in an initial 100% field capacity (FC) after sowing and subsequent constant 80% FC for W variants, and irrigation interruption from day 3 to day 16 for the D variants and posterior rewatering for 4 days. Blue arrows represent the watering regime and red arrow the stop of the irrigation moment in the D variant. **(B)** Changes in projected canopy height (in pixels) and **(C)** side view images of the D and W variants from the three independent replicates along the experiment. Asterisks indicate the significance level relative to the control variant after Kruskal-Wallis test. *P≤ 0.05.

For studying water stress response and recovery capacity in barley at a population level, we performed a final **Experiment 3** (**Supplementary Table S1**). In this case, the projected canopy height of five randomly distributed trays containing two plastic containers; a water-stressed variant (D, left) and a well-watered variant (W, right) with 50 plants each (with a final number of 500 barely seedlings), was recorded over the time-course of 15 days (**Figure 1** and **Supplementary Figure S1**). Three biological replicates with five plants (3 × 5 = 15 plants) were taken from two randomly selected trays in each variant (D and W) and then, they were used for validation and reproducibility of the method. The three remaining trays were collected at the end of the rewatering period. First of all, we determined the substrate water content of all the trays during the experiment. For the first 6 days, the substrate water content of the irrigated and non-irrigated variants was very similar, with no differences between treatments (**Figure 3A**). At day 9, the substrate water content of non-irrigated variants started to decrease with significant differences compared to W variants (*P* < 0.05). At day 15, the substrate water content of the D variant reached 65%. In this point, the containers with the D variant were rewatered to 100% full capacity (**Figure 3A**).

**Figure 4:**
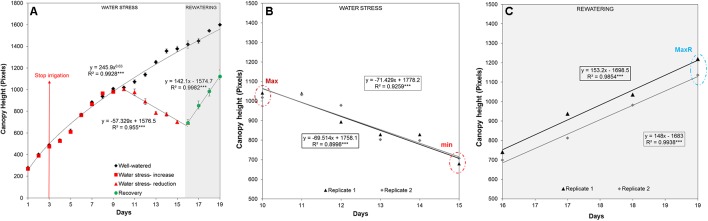
Linear curve of the projected canopy height and the extracted traits. **(A)** The average canopy height, regression curve and significance calculated from three independent trays (with 50 plants each) growing under water deficit conditions for 13 days (with the endpoint at day 16) and with subsequent rewatering for 4 days (with the endpoint at day 19). **(B)** Max is the maximum and Min the minimum canopy height reached by the stressed plants from replicate 1 or 2 (n = 50) under water deficit conditions, and the slope of the linear model curve is shown. **(C)** MaxR is the maximum canopy height from replicate 1 or 2 (n = 50) attained after 4 days of rewatering, and the slope of the line (SlopeR) is also shown in the equation.

The automated RGB imaging yielded curves with the same pattern for the five independent trays until the end of the water deficit period (**Supplementary Figure 1**) and for three trays through the whole experiment (**Figures 3B, C**). For the first 7 days after water withdrawal, the stressed plants retained projected canopy height kinetics similar to the well-watered plants, but they were significantly reduced from day 11 to day 15 (**Figure 3B** and **Supplementary Table S2**). After rewatering, the D variant from each independent tray similarly recovered the projected canopy height but the values were still significantly smaller than the W variants (**Figure 3B** and **Supplementary Table S2**). From the obtained dynamic changes in the projected canopy height of the barley seedling population, we created a model curve different for the stressed and non-stressed variants, as represented in **Figure 4A**. From each curve, we extracted several traits; the maximum canopy height on the days when watered and non-watered curves separated (Max), water loss as the slope of line obtained for the reduction in canopy height, the minimum canopy height for the water stress period (Min), the water recovery capacity as the slope of the line obtained for the increase of canopy height after rewatering, and the maximum canopy height after rewatering (MaxR). As an example of the potential use of our bioassay, we calculated the slope of the line for the water deficit period and after rewatering from the two trays selected for the posterior manual measurements (**Figures 4B, C**). In all linear curves, a highly significant correlation (*R* ^2^ > 0.90; *P* < 0.001) was obtained for both growth conditions. Together with the slopes, the Max, Min, and MaxR are shown in **Table 1**; similar values were obtained in both replicates for these traits. These results confirmed the reproducibility of the method, which was later validated using destructive parameters.

**Table 1:** Traits related to water deficit and recovery. Traits were extracted from the linear regression of the canopy height in two randomly selected trays.

	Water stress				Rewatering	
	Day	Max	min	Slope	MaxR	SlopeR
**Tray 1**	8	1041	680	-69.514	1218	153.2
**Tray 2**	8	1018	707	-71.429	1136	148

Day is the day when treated and not treated plants are different; Max is the maximal canopy height reached in the stressed plants under drought; min is the minimal canopy height at the end of the drought period; Slope of the linear curve from the canopy height in the drought period; MaxR is the maximal canopy height reached in the stressed plants after rewatering; and SlopeR of the linear curve from the canopy height in the rewatering period.

**Figure 5:**
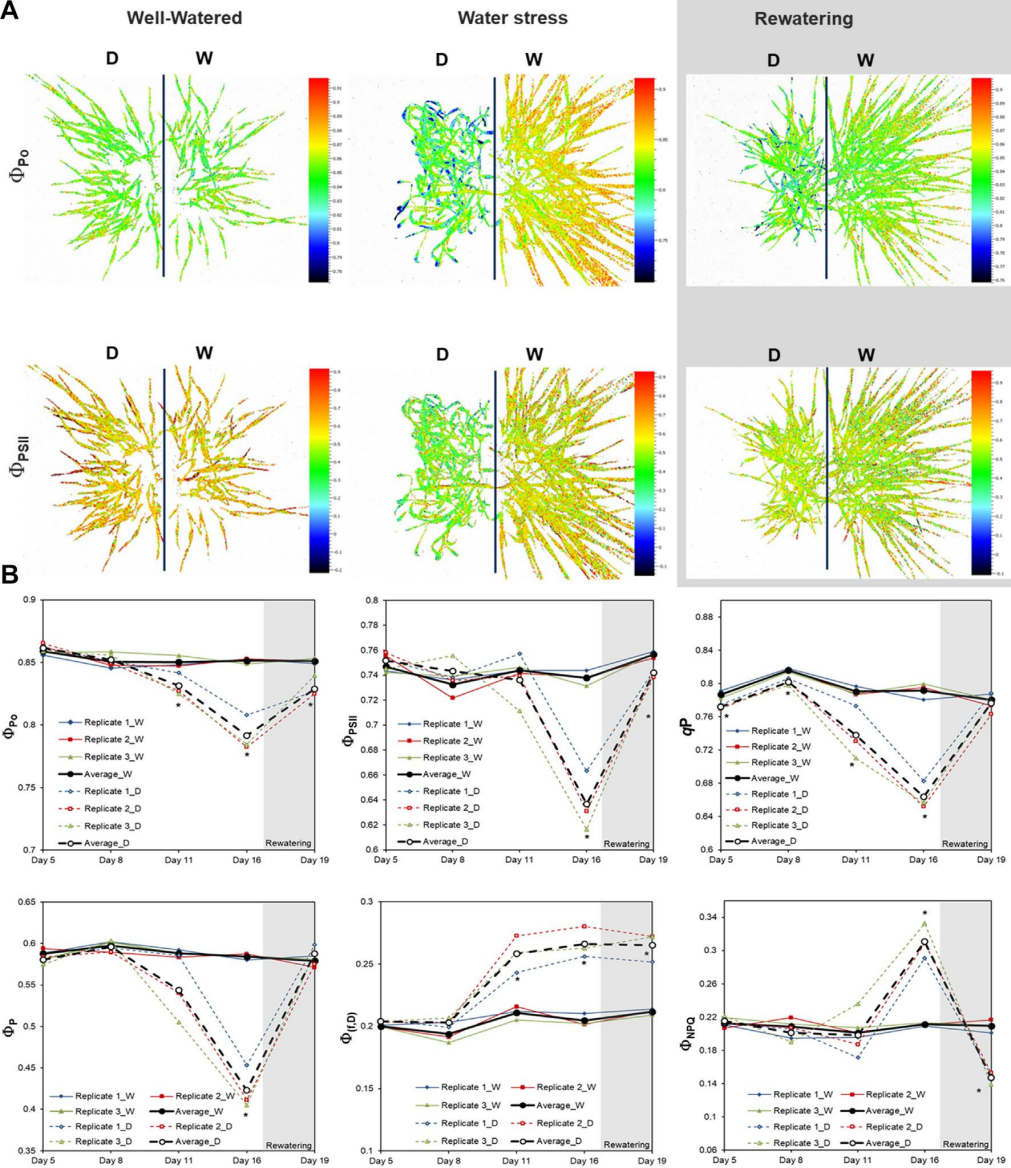
Variation in chlorophyll parameters in barley (*Hordeum vulgare*) seedlings grown under water stress conditions and after subsequent rewatering. **(A)** Imaging of chlorophyll fluorescence (Φ_Po_ and Φ_PSII_) in barley seedlings under well-water, water stress, and rewatering. Stressed and non-stressed plants labeled as D (left) and W (right), respectively. **(B)** Chlorophyll fluorescence parameters from three independent trays (Barley 1, 2, and 3) (n = 50) and the average values. D and W variants are represented by discontinuous and continuous lines, respectively. Statistical analyses were performed *via* ANOVA. Asterisks indicate the significance level relative to the control variant; *P≤ 0.05.

Our phenotyping system is also equipped with a FluorCam unit for the analysis of Chl fluorescence parameters. In our study it was clear that water deficit significantly changed all the Chl fluorescence parameters, albeit with different dynamics and intensity (**Figures 5A, B**). Some parameters responded quickly and had already changed by day 11, e.g., Φ_Po_ and *q*P had decreased, and Φ_(f,D)_ had increased. Other parameters, such as decreasing Φ_PSII_ and Φ_P_, and increasing Φ_NPQ_, had occurred by day 16 as late stress response parameters (**Figure 5B**). After rewatering, the stressed variants recovered to control values for *q*P and Φ_P_. However, Φ_Po_, Φ_PSII_, Φ_(f,D)_ and Φ_NPQ_ maintained significant differences compared with the well-watered plants (**Figure 5B**). The results indicated that these last four parameters were potential indicators for evaluating the recovery capacity of plants, at least in barley.

### Validation and Reproducibility of the Phenotyping Method

#### (A) The Reproducibility of Morphometric and Physiological Parameters Between Biological Replicates and Trays Confirmed the Reliability of the Method

**Figure 6:**
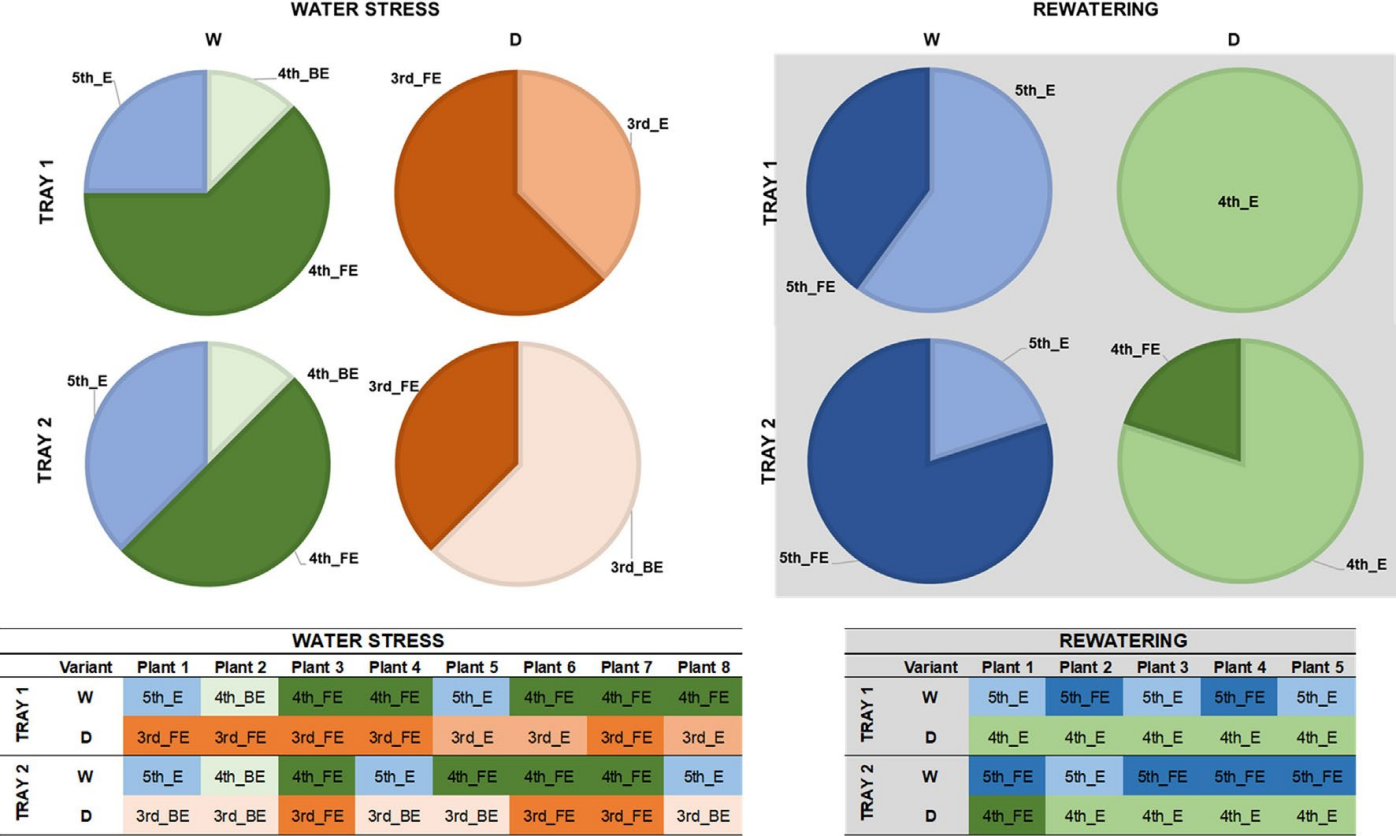
Developmental stages of barley seedlings (*Hordeum vulgare*) under water stress and after subsequent rewatering. Developmental stages of leaves in stressed (D) and non-stressed (W) plants from two independent trays (1 or 2) at the end of the water stress period (n = 8) (Left panel) and after rewatering (n = 5) (Right panel).

We performed a manual evaluation of several morphological and physiological parameters in two independent trays to integrate and validate our results obtained by imaged-based measurements. First, we evaluated the developmental stage of the plant population by analyzing the last emerging leaf in eight and five randomly selected plants per variant at the end of the water stress period and rewatering, respectively (**Figure 6**). We observed differences among variants (treated and non-treated) and not between trays. Whereas in the watered control at day 16 the fifth leaf had appeared, the stressed plants were still expanding the third leaf at this point of the assay (**Figure 6**). After rewatering, the differences were reduced to one leaf less, with the fourth and fifth leaf in expansion for stressed and non-stressed plants, respectively (**Figure 6**).

**Figure 7:**
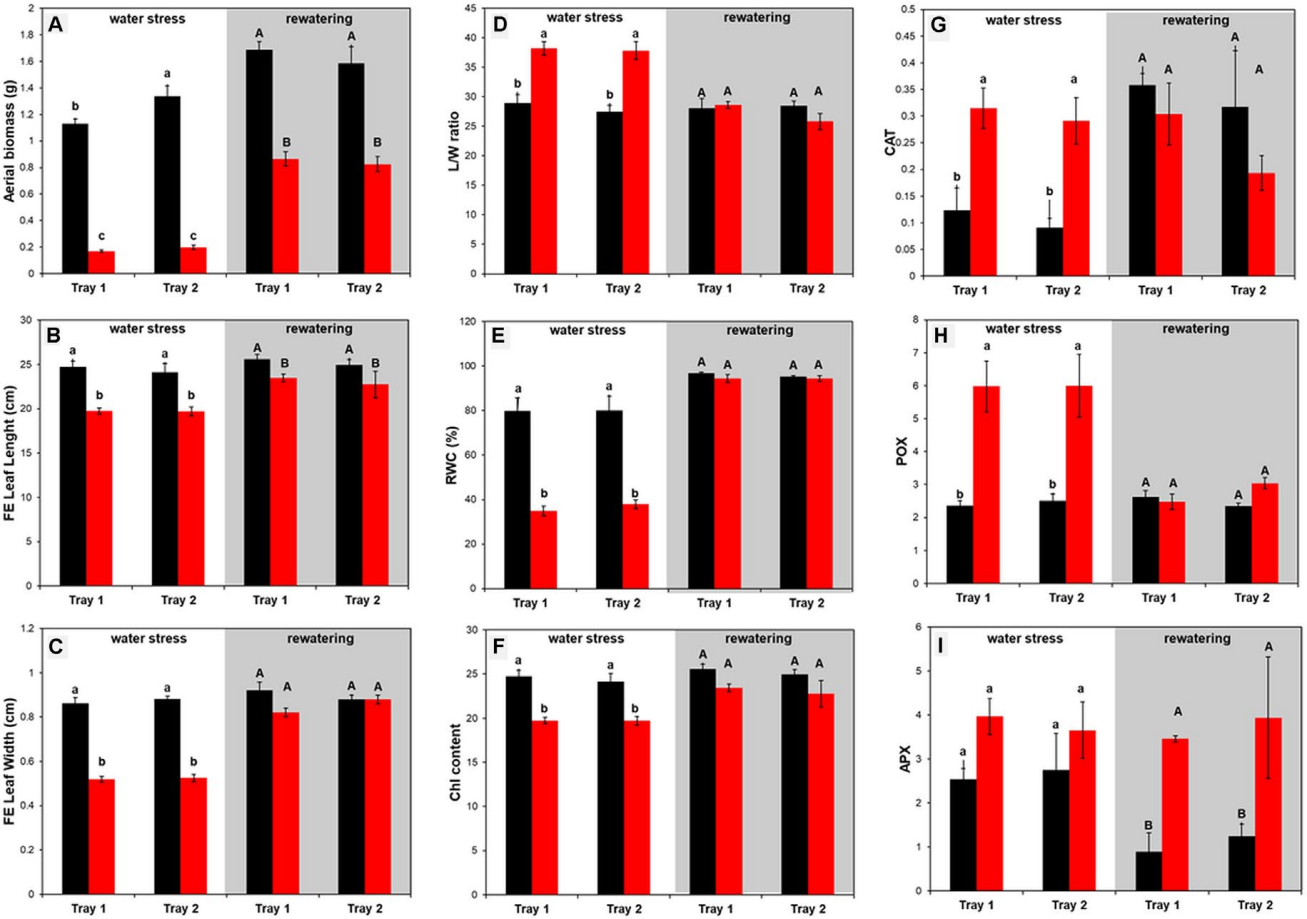
Morphometric and physiological changes in barley (*Hordeum vulgare*) seedlings under water stress conditions and after subsequent rewatering. **(A)** Aerial biomass (FW, g), **(B)** leaf length (cm) and **(C)** width (cm) of the last fully expanded (FE) leaf, **(D)** the ratio between length and width, **(E)** the relative water content (%), **(F)** the index of the chlorophyll content, and the activity of the antioxidative enzymes **(G)** guaiacol peroxidase (POX, µmol s_–1_ mg_–1_ protein), **(H)** catalase (CAT, mmol s_–1_ mg_–1_ protein) and **(I)** ascorbate peroxidase (APX, µmol s_–1_ mg_–1_ protein), in stressed (D, color bars) and non-stressed (W, black bars) barley seedlings from two independent trays at the end of the water stress period (n = 8) and after subsequent rewatering (n = 5). Three independent pools containing five plants each were used for the quantification of the antioxidant enzyme activity. Different letters mean significant differences according to Tukey HSD test after ANOVA.

The aerial biomass (FW, g) of full expanded (FE) leaves, the length and width (cm) and the ratio between length and width of eight plants per variant and tray were individually collected and measured at the end the water stress period and after rewatering, respectively (**Figures 7A-C**). In almost all the cases, the changes were due to the treatment and not because of the tray position effect (**Supplementary Table S3**). Regarding biomass, the seedlings from the D variant were significantly smaller than the W plants in both trays (**Figure 7A**). After recovery, D plants were still only half the weight of the well-watered variants (**Figure 7A**). When the length and width of the last fully expanded leaf were evaluated, we observed that the seedlings from the same variant showed the same profile for both traits (**Figures 7B, C**); the length and width were significantly reduced under water stress conditions. After rewatering, only the width recovered to control values (**Figure 7C**), affecting the length/width ratio (**Figure 7D**).

There was also no effect of the tray position recorded for the RWC and total Chl content between the trays (**Supplementary Table S3**).The water deficit treatment caused 50% decrease in plant RWC, and rewatering returned the plant water status to 95% in only 4 days (day 19) (**Figure 7E**). Total Chl content showed a similar pattern (**Figure 7F**). The values were significantly lower in stressed plants than in the controls, indicating that the Chl content and N status of the plants were significantly affected by water stress conditions but were recovered after 4 days of rewatering (**Figure 7F**).

Altogether, the high reproducibility of the individual biometric and physiological parameters among the trays corroborated the reproducibility of the method for studying water deficit and recovery capacity at least in barley cv. Golden Promise.

#### (B) The Reproducibility of the Antioxidative Response Between Trays Validated the Method to Study Water Deficit and Recovery in Barley Population

It is well known that plants activate their antioxidative machinery as a response to stress. Hence, we quantified the activity of the three antioxidant enzymes CAT, POX, and APX (**Figures 7G-I**). The same profile was observed between trays as stress response and subsequent recovery (**Figures 7G-I**, **Supplementary Table S3**). Interestingly, only CAT and POX increased significantly in the stressed variants, and then recovered to control values after rewatering (**Figures 7G, H**). In contrast, APX kept increasing during the recovery period, resulting in values four times higher in the stressed seedlings than in the controls (**Figure 7I**). These results indicated that each type of antioxidant enzyme plays a different role in the barley cv. Golden Promise stress response.

#### (C) Different Free AAs and PAs Regulated Plant Stress Response and Recovery

**Figure 8:**
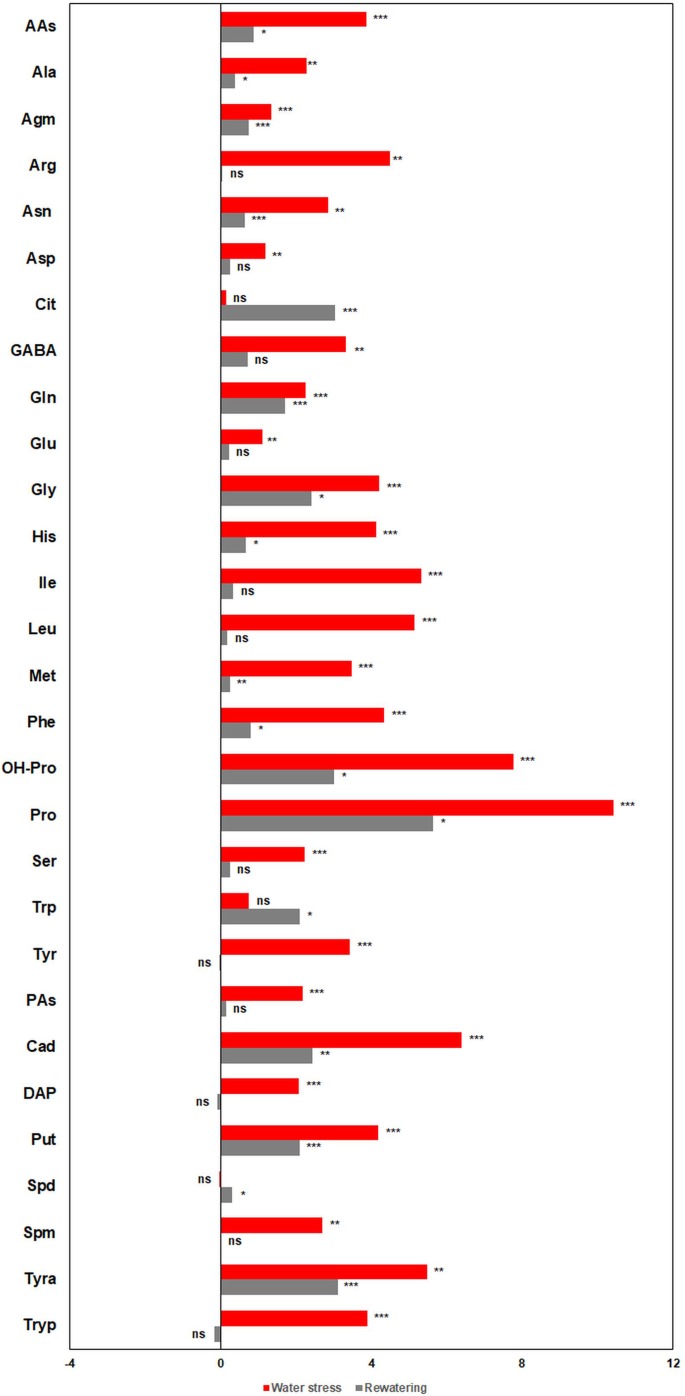
Metabolic profiles of barley (*Hordeum vulgare*) seedlings under water deficit and after subsequent rewatering. Fold changes (presented as log_2_ ratio) in the content (pmol mg^-1^ DW) of free polyamines (PAs) and amino acids (AAs) between stressed (D) and non-stressed (W) barley seedlings (three independent pools containing five plants from two independent trays, n = 6) at the end of the stress period (red bars) and after subsequent rewatering (gray bars).

Free PA and AAs are typical metabolites involved in the plant water stress response (Podlešáková et al., 2019) (**Figure 8**). As expected, both groups of metabolites accumulated significantly under stress, and the profile of the fold change between treated and non-treated plants was similar (**Figure 8**).

Water deficit induced a significant accumulation of AAs (pmol mg^−1^ DW) (**Figure 8** and **Supplementary Table S4**), with the highest increases in Pro and OH-Pro. After rewatering, many of the AAs kept significantly higher in the stressed plants than in the controls, except for Arg and GABA which did not show significant differences between treatments (**Figure 8**). Interestingly, at this timepoint, the accumulation of Cit in stressed plants was 11.7-fold (log_2_ = 3.55), which is higher compared with the control levels (**Figure 8**).

For the PAs, Tyra and Cad were the most accumulated compounds, and Spd was the only PA that did not change in stressed plants compared with the non-stressed ones (**Figure 8** and **Supplementary Table S4**). After rewatering, water-stressed plants kept higher content of Tyra, Cad, and Put compared with the well-watered plants, Spd accumulated to higher levels, whereas no significant differences between variants were found in the case of DAP, Spm, and Tryp (**Figure 8** and **Supplementary Table S4**). Based on these results, we could classify the metabolites into three groups, water stress-related compounds (e.g. Pro, OH-Pro, Tryp, and Spm), a group involved in the recovery of the plants after rewatering (e.g. Cit and Spd), or those compounds that were in high levels in both conditions (e.g., Cad, Tyra, or Put).

### Data Analysis—Multivariate Statistical Analysis for Understanding the Physiological Basis of the Image-Derived Traits and Water Stress Response in Barley Populations

**Figure 9:**
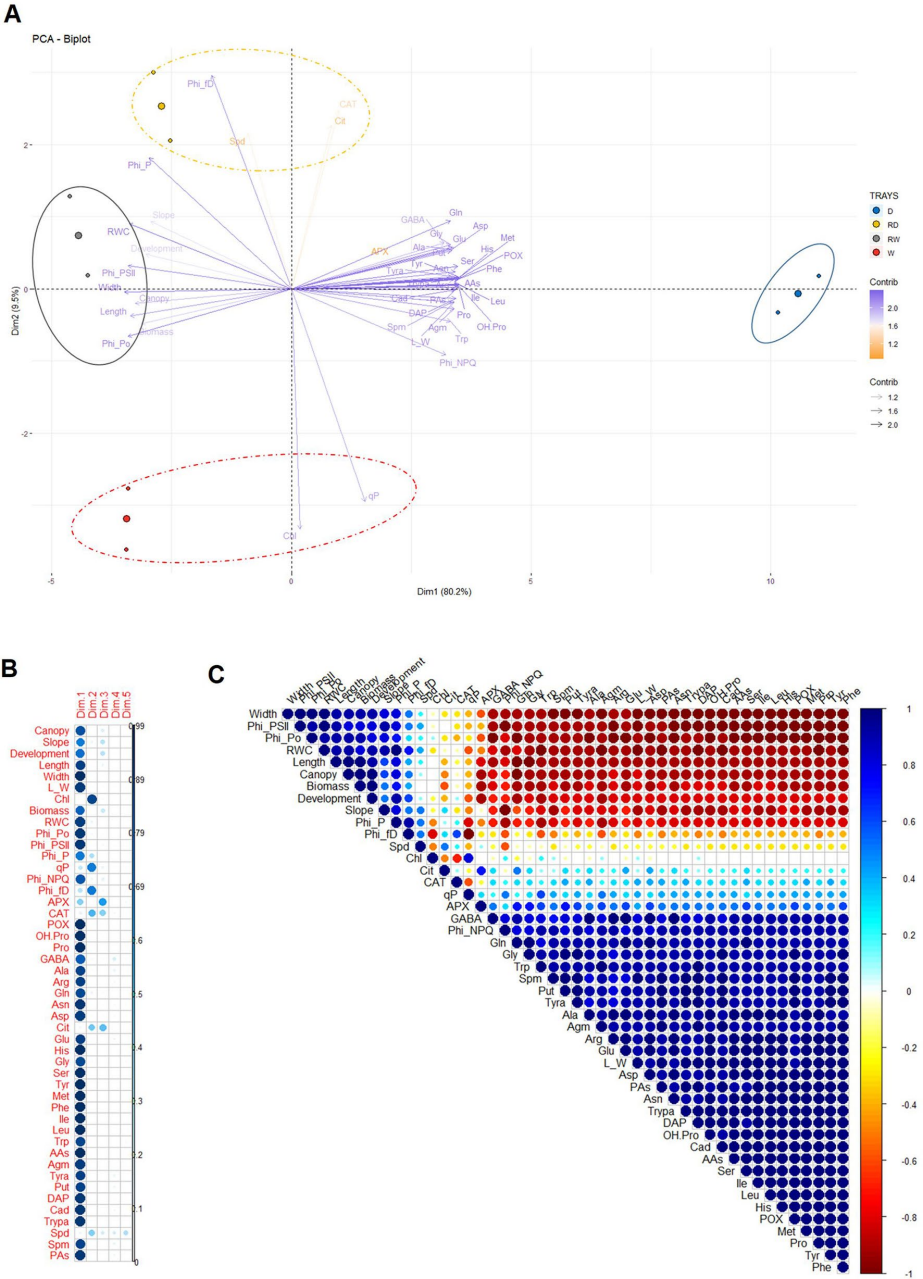
Multivariate statistical analyses of the traits in barley seedlings related to the water stress response and subsequent rewatering. **(A)** Principal component (PC) analysis **(B)** contribution of the loadings to each PC (Dim) and **(C)** a correlation matrix of 47 variables, including 15 traits and 32 biochemical metabolites obtained from different biological replicates of two independent trays with barley seedlings at the end of the water stress period and after subsequent rewatering.

To integrate all parameters measured and confirm the reliability of the traits derived from our method based on the canopy height of barley populations, we performed a PCA in which trays 1 and 2 were evaluated for two different growth regimens, water-stressed (D) and well-watered (W) plants, at two timepoints, at the end of the stress period or after 4 days of rewatering (RD and RW, respectively). To facilitate visualization, the results were projected onto a biplot representing the scores (variants) and the loadings (analyzed traits) (**Figure 9A**). The first two principal components (PC1 and PC2), which together captured 89.7% of the variance, explained the experimental model almost completely (**Supplementary Figure S2**). We could see that the trays with the same variant and timepoint were closely located. PC1 accounted for 80.2% of the total variation, and included almost all the traits and metabolites, except Chl, *q*P, Φ_(f,D)_ (Phi_f,D), APX, CAT, Cit, and Spd. The accumulation of metabolites was positively correlated with the water deficit conditions (D) (**Figure 9A**). In contrast, they showed a negative correlation with the controls from the water stress and rewatering period, which were positively correlated with the canopy height, slope, development, Φ_Po_ (Phi_Po), Φ_PSII_ (Phi_PSII), Φ_P_ (Phi_P), biomass, leaf length and width, and RWC. PC2 captured an additional 9.7% of the total variance, and was positively dominated by CAT, Cit, and Spd in the rewatered trays (RD) (**Figures 9A, B**). This analysis demonstrated the reproducibility of the measurements performed, as the independent trays were grouped together in all analyzed situations. In addition, the separation of the loadings allowed us to identify possible traits related to water stress and rewatering responses in the barley cultivar used in this study.

Finally, to reduce the number of possible traits observed in the PCA, we performed a linear correlation across all traits. The outcome helped us to (a) validate our non-invasive method with other traditionally invasive parameters and metabolites and (b) determine the collinearity among them for identifying the most representative traits of the water deficit response. For better visualization of the results, we created a correlation matrix and four scatter plots (**Figure 9B** and **Supplementary Figures S3**–**S6**). The correlation matrix was constructed based on Pearson correlation coefficients, which is represented by circles with different intensity colors and sizes, blue (positive) or red (negative) (**Figure 9C**). Similarly, we prepared four scatter plot matrices from the most correlated traits that included sloped linear regressions, Pearson correlation coefficients, and significance (**Supplementary Figures S3**–**S6**). We found that canopy height had the strongest positive correlation with aerial biomass and leaf length, with an *R* *_2_* of 0.99 and 0.98 (*P* ^***^ ≤ 0.001), respectively (**Figure 9C** and **Supplementary Figure S3**). The correlation of canopy height versus RWC was also positive (*R* *_2_* = 0.88, P** ≤ 0.01), showing that this non-invasive parameter integrated both the growth and water status of the plants. Canopy height was positively correlated with Φ_Po_, Φ_PSII_, and Φ_P_, and negatively with Φ_NPQ_, APX, and POX, several AAs and PAs, except for Cit and Spd (**Figure 9C** and **Supplementary Figures S3**–**S5**). Interestingly, these two compounds showed a very different response compared with the rest of the metabolites and presented a significant correlation from each other (*R* *_2_* = 0.77, P* ≤ 0.05) (**Supplementary Figure S6**). Besides, Cit showed linear correlation with the antioxidant enzyme APX (*R* *_2_* = 0.72, P* ≤ 0.05). Another phenotype-derived trait, the slope of the curves, did not show any linear correlation with the morphometric traits related to canopy height and was directly correlated with Φ_PSII_ (*R* *_2_* = 0.92, *P* ^**^ ≤ 0.01) and inversely with Φ_NPQ_ (*R* *_2_* = 0.97, *P* ^***^ ≤ 0.01) (**Supplementary Figure S4**). These results suggested that the slope or plant water turgor was influenced by the light intensity and showed that the side-view traits of canopy height and slope provided additional useful information for studying and understanding the water stress response of barley populations.

### Demonstration of the Method Applicability

**Figure 10:**
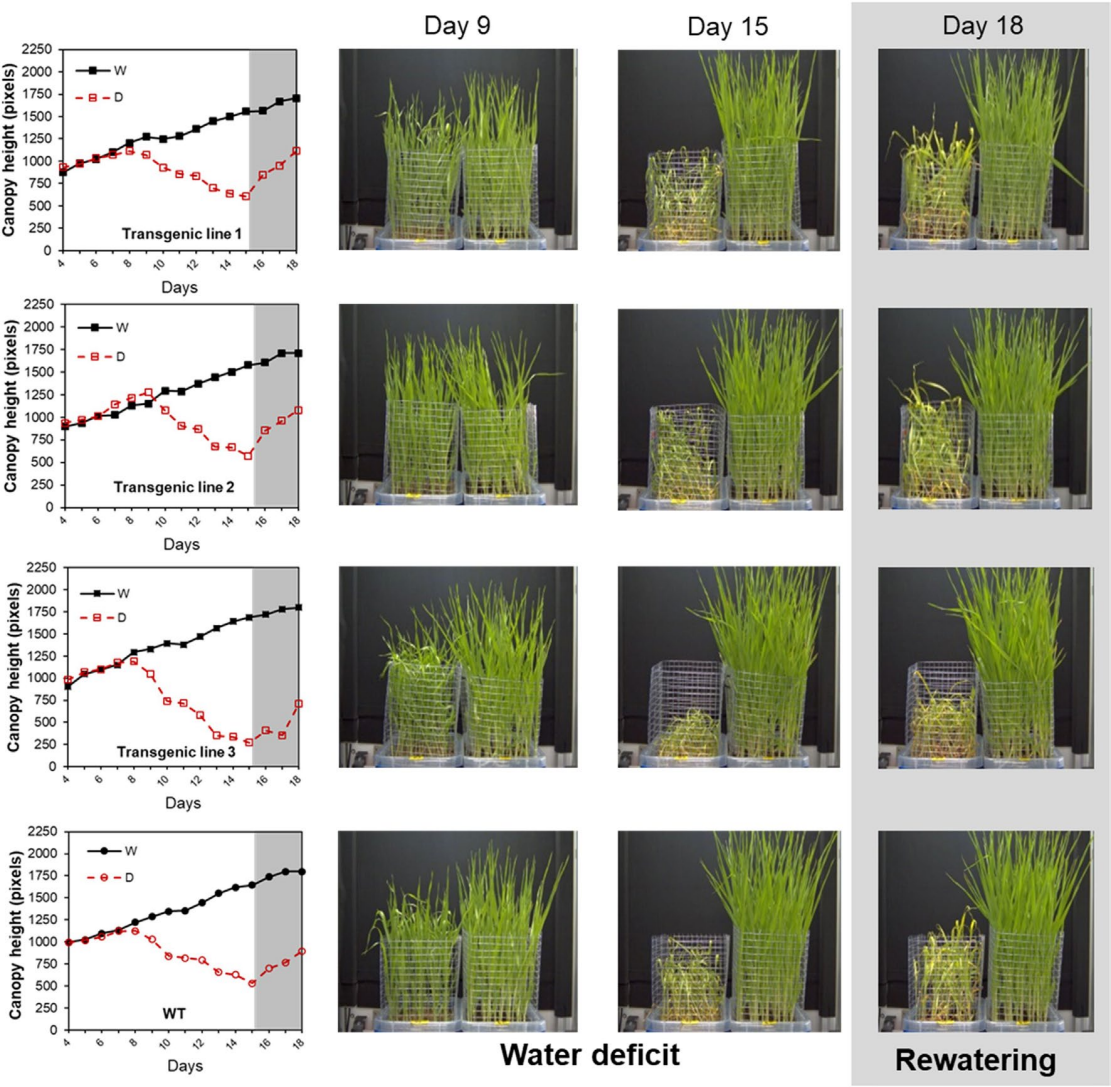
Projected canopy height in barley seedlings from different transgenic lines growing under water stress conditions with subsequent rewatering. Changes in projected canopy height (in pixels) and side view images of the D (left) and W (right) variants of three transgenic lines with silencing transgene A (transgenic lines 1, 2, and 3) and wt during a water stress period and subsequent rewatering.

To demonstrate the method applicability, wt and three different transgenic lines with different levels of a gene A silencing were phenotyped under water stress conditions and after rewatering. As presented further, the transgenic lines 1, 2, and 3 with the 1, 10 and 4 T-DNA insertions, respectively, reacted differently to the water stress and rewatering. Interestingly, our method was sensitive enough to discriminate between lower and higher sensitive lines and recorded different behaviors of the individual lines pointing to the number of T-DNA insertions as the determinant of the lines’ performance. As shown in **Supplementary Figure S7**, canopy height and aerial biomass presented a highly significant correlation. Different growth curves were obtained for each line; we observed that plants of the transgenic line 2 were able to maintain optimum growth under water stress conditions up to day 9, whereas the rest of the lines have already stopped growing at day 7 (**Figure 10**). After rewatering, the plants from this line and line 1 recovered faster compared with the wt and the transgenic line 3 that showed worse performance. We also analyzed the content of PAs in these barley lines to corroborate their involvement regulating plant stress response and/or recovery capacity (**Supplementary Figure S8**). The lines have a clear alteration in the PA metabolism compared to wt. In general, the transgenic lines accumulated less Put and more Tryp and DAP under water stress conditions. After rewatering, they reduced the total PA content almost to control; however, some metabolites, such as Cad and Agm, were maintained high, especially in the genotypes with lower recovery capacity, wt, and Line 3 (**Supplementary Figure S9A**).

**Figure 11:**
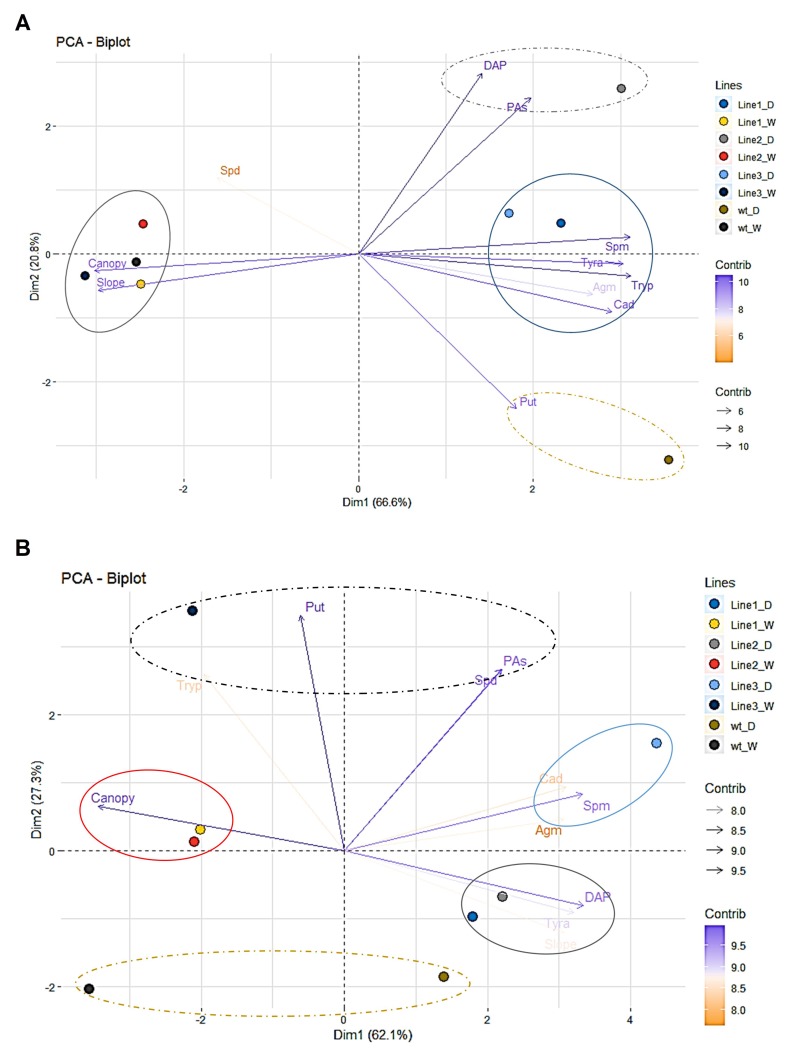
Multivariate statistical analyses of the traits in barley seedlings from different transgenic lines related to the water stress response and subsequent rewatering. Principal component (PC = Dim) analysis at the end of the water stress period **(A)** and after subsequent rewatering **(B)**.

To clarify the correlation between the PA content and the phenotype of the barley plants, we performed two PC analyses, under water stress conditions and after rewatering (**Figures 11A, B**). The PC1 (Dim1), which accounted for 66.6% of the total variation separated the lines in two groups, well-watered and stressed (**Supplementary Figure S9A**). The PC2 that captured an additional 20.8% of the total variance was mainly represented by the loadings DAP, Put, and PAs (**Supplementary Figure S9B**), which separated the wt and the transgenic line 2 (**Figure 11A**). In this case, the higher tolerance of the transgenic line 2 was correlated to the higher content of PAs and mainly DAP, whereas the wt mainly accumulated Put. However, no DAP neither Put presented lineal correlation with the canopy height or slope, which were negatively correlated with other PAs such as Tryp, Tyra, Spm, or Cad (**Supplementary Figures S9C, D**). The additional three different groups were distributed in the PC analysis analyzing the rewatering. Whereas the line 3 that showed the lowest capacity of recovery was positively correlated with the levels of Agm, Cad, and Spm, lines 1 and 2 showed higher slope, and DAP and Tyra levels (**Figure 11B**, **Supplementary Figure S10**). Altogether, we showed that the transgenic line 2 with the highest number of transgene integrations for the silencing cassette presented the higher stress tolerance and recovery capacity. These results demonstrated applicability of our method pointing to the canopy height and PA analysis as sufficient markers for discrimination between water stress sensitive and tolerant barley lines and their recovery capacity.

## Discussion

Indoor phenotyping methods enable the evaluation of various traits from a large number of plants in a fast and non-invasive manner. Routinely, experiments are performed in controlled conditions mimicking environmental conditions. However, an effective translation of indoor simulations to what plants experience in the field is not straight-forward because of the complexity and variability of the field conditions, which complicate data analysis and interpretation (Araus and Cairns, 2014). As reviewed by Poorter et al. (2016), it is necessary to develop new protocols that can adjust growth conditions to be closer to field conditions. A common problem is the use of high relative humidity (RH) in protocols for studying water deficit (Junker et al., 2015; Nosalewicz et al., 2016), whereas in the field, water stress is frequently associated with low RH values. We have optimized our method for studying water deficit in barley at a RH of 40%, which is closer to real conditions and thus accelerates the stress response. Another limitation in the translation from the laboratory to the field is the method of growing the plants in different spatial conditions. Indoor phenotyping traditionally analyzes the plant growth in individual pots (Honsdorf et al., 2014; Cabrera-Bosquet et al., 2016; Poorter et al., 2016), whereas in the field, plants grow to form a canopy, competing with neighbors for above- and below-ground resources. To form the canopy, in our method, we planted 50 barley seedlings in the same plastic container (**Figure 1**), reaching a final density of 1000 plants m^–2^ instead of the 20 to 50 plants m^–2^ normally used in indoor phenotyping (Poorter et al., 2016). When plants are grown in individual pots, image analysis allows to dissect plants as individuals, because they are not masking each other (Chen et al., 2014; Honsdorf et al., 2014). Plants grown in a dense canopy overlap the leaves, making it almost impossible to extract projections of single plants. We overcame this by analysis of whole canopies instead of individuals. Using side-view RGB images and developing a software routine, we can estimate the projected canopy height of the barley population (**Figures 1** and **4**). This provided indirect information about several invasive parameters, such as biomass, leaf length and width, and RWC (**Figure 9** and **Supplementary Figures S3**–**S6**), which are important manually measurements for studying barley populations in water stress conditions. Chen et al. (2014) previously described a similar phenotyping setup with a water stress phase followed by a re-watering phase. They also showed that the plant height was highly correlated with the destructive measured shoot biomass. However, they evaluated single plants and used very sophisticated and expensive sensors, such as FluorCam and NIR. Later on, Neumann et al. (2015) showed that the evaluation of derived traits from the obtained curve of the barley-accumulated biomass is an efficient approach to characterize different lines under water stress condition and rewatering using a model obtained from plants grown individually. To our knowledge, our indoor phenotyping approach is the first that uses projected canopy height in populations, representing overall growth conditions that are more similar to those that plants experience in the field, especially when compared with single pot-single plant studies. Besides, we also showed that from each obtained canopy height curve, it is possible to extract more traits, such as the slope of the obtained line for the water stress and recovery period (**Figure 4** and **Table 1**). In addition, a simple and cheap RGB camera is enough to analyze the population canopy height, making this phenotyping method adaptable to other commercial or home-built systems.

The reliability and repeatability of the method was validated by two independent experiments under control conditions (**Figure 2**), and under water deficit with subsequent rewatering in randomly distributed trays (**Figure 3** and **Supplementary Figure S1**), showing similar behaviors for 47 analyzed traits and metabolites (**Figures 3**–**9**). Regarding Chl-related traits, water stress reduced the total Chl content in barley seedlings (**Figure 7F**) and the maximum quantum yield in the dark- and light-adapted stages Φ_Po_ and Φ_PSII_, and increased the regulatory and non-regulatory dissipation processed reflected in Φ_NPQ_ and Φ_(f,D)_, respectively (**Figure 5**), to ameliorate photoinhibition and protect the photosynthetic apparatus (Xu et al., 1999). Similar observations have been reported in many studies of the water stress response of barley (Li et al., 2006; Filek et al., 2015). However, we observed that whereas some parameters changed in parallel with the projected canopy height, others changed at a later stage. For example, the decrease in Φ_PSII_ happened at the end of the water stress period (**Figure 5B**), as observed by Berger et al. (2010), disabling it as a possible marker of early water stress response in barley. However, *q*P was a fast response fluorescence parameter (reduced from day 5) (**Figure 5B**) and positively correlated to Chl (**Supplementary Figure S3**). Optimal utilization of photochemical energy in carbon metabolism is characterized by high *q*P values. When light absorption exceeded the requirement by carbon metabolism, the *q*P declines. Thus, it serves as a good indicator of “light stress” (Gallé and Flexas, 2010). However, the fluctuations of this parameter have been described as cultivar dependent at least in wheat (Wang et al., 2016). Altogether, these results suggested that under our growth conditions, the water-stressed plants might suffer photoinhibition and that *q*P could be a good trait for estimating the photosynthetic status of barley plants during water stress conditions and subsequent recovery.

Water deficit increases the production of reactive oxygen species (ROS) in plants that stimulate antioxidant enzymes to counteract the oxidative damage and detoxify ROS. Similarly, in our work, the barley seedlings increased the antioxidant enzyme activity of CAT and POX in stressed plants compared with the controls (**Figures 7G, H**). However, APX only increased significantly after the rewatering. It has been described that APX enhanced tolerance under water deficit in many plant species, but the increased expression of *APXs* varied in different developmental stages, among species and type of stress (reviewed by Pandey et al., 2017). Another study demonstrated that a simple pre-treatment with a stressor can induce an increase in the activity of this enzyme, protecting the plants against future stress events, a process traditionally called hardening (Hsu and Kao, 2007). These assumptions suggested that the high APX activity in the stressed plants after recovery could be related to the developmental stage (4^th^ leaf, **Figure 6**) and/or a fast mechanism to reduce ROS.

Another strategy of plants to cope with drought-induced stress is the synthesis and/or accumulation of compatible solutes (e.g. AAs), which can be involved in ROS scavenging and/or perform as signal molecules (Urano et al., 2010). As revealed by metabolite profiling, most of the AAs accumulated in barley under water stress conditions (**Figure 8A**), especially Pro and OH-Pro. Pro was described in 1972 (Singh et al., 1972) as a useful marker for screening the physiological status of plants grown under water deficit conditions. In our work, even when the rewatered plants recovered their RWC, they maintained both Pro and OH-Pro levels above the control values (**Figure 8B**). This was already described in barley (Reddy et al., 2004), as well as in other species (De Diego et al., 2013; Zhang et al., 2018). It is worth mentioning that Cit was the only AA that kept increased levels after rewatering (**Figure 8**). Although there are only few works reporting the possible role of Cit in the plant stress response, Akashi et al. (2001) demonstrated that Cit was a more effective hydroxyl radical scavenger than other compatible solutes, such as Pro or Mannitol, and it can effectively protect DNA and enzymes from oxidative injuries. Thus, Cit could accelerate the recovery of the stressed plants by reducing the stress-induced oxidative damage, being an interesting metabolite for evaluating acclimation to water stress in barley (**Figure 9A**).

Regarding PAs, Put, Spd, and Spm are usually accumulated under stress conditions (Podlešáková et al., 2019). However, in our study, Cad, DAP, Tryp, and Tyra were the major contributors to the water stress response (**Figure 8** and **Supplementary Figure S8**). In fact, Cad, DAP, and Tyra, but not Tryp, remained at higher levels in the stressed plants even after rewatering (**Figure 11**). The conversion of Spd to DAP is a stress response that can control ROS (Bitrián et al., 2012; Liu et al., 2015). DAP accumulation during water deficit and recovery could also be a mechanism for modulating membrane electrical and ion transportation properties and for controlling stomata closure, counteracting the action of ABA (Jammes et al., 2014). Cad is synthesized by an independent pathway through lysine catabolism, and its accumulation has been reported in other species under stress conditions (Jancewicz et al., 2016). Although the role of Cad in the stress response is still unclear, it has been recently shown that this PA displays anti-senescence activity (Tomar et al., 2013), suggesting that it is another important ROS-modulating compound and possible plant growth regulator (Jancewicz et al., 2016). However, when the levels of Cad were quantified in transgenic lines with different stress response, the accumulation was more indicator of stress level rather than tolerance (**Figure 11**). Concerning Tyra, there are few studies showing its accumulation in plants under stress (Aziz et al., 1999; Lehmann and Pollmann, 2009). Aziz et al. (1998) have shown that Tyra can regulate Pro production. In addition, Wang et al. (2019) pointed to Tyra as biomarker of salt stress tolerance in barley. Transgenic lines also accumulated higher levels of Tryp than wt under water stress condition (**Figure 11**). Previous studies also showed the accumulation of Tryp as barley defense against UV and biotic stress (Miyagawa et al., 2014). It has been demonstrated that in plants, the catabolism of Tryp can produce serotonin and melatonin as plant stress response (Hayashi et al., 2016). However, the tolerance and sensitivity in the genotypes must be more dependent of their interconversion (including synthesis and catabolism) and the crosstalk between their related function rather than a concrete one. Finally, in our study, highly significant correlations (*P* ^***^ ≤ 0.001) existed between Put, Spm, DAP, Tryp, Tyra or Cad, and Pro (**Supplementary Figure S6**), results that supported the existence of a relevant crosstalk between PAs and Pro regulating plant stress response (Podlešáková et al., 2019). In addition, the results obtained in the characterization of transgenic lines with silenced transgene A demonstrated that quantification of PAs is enough to define the level of stress in this barley genotype under water stress and recovery (**Figure 11** and **Supplementary Figure S9**–**S10**).

In conclusion, we have designed, optimized, and validated a non-invasive image-based method for automated high-throughput screening of potential water stress tolerance phenotypes using projected canopy height in barley [an additional movie file shows this in more detail (see **Supplementary File S1**)]. Using multivariate statistical methods, we have also identified new metabolites involved in stress response and recovery using different transgenic lines of barley. As shown here, projected canopy height is sensitive trait that truly reflects other studied morphological and physiological parameters and metabolites. It represents very informative, simple, and robust trait that does not require an expensive sensor and, hence, is suitable to be used in low-cost systems using single RGB imaging. It can be also seen as easy to understand and self-speaking trait, since the reduction of plant growth and loss of turgor are traditional traits to characterize the water stress response of the plants. Besides, we demonstrated that the simple analysis of canopy height combined with quantification of PAs bring enough data to define the plant stress strategy. For this reason, our method can be used to study the mechanisms involved in the water deficit response and recovery capacity of crops, such as barley. We believe that it has high potential to be integrated into breeding programs for fast screening and identification of stress-tolerant genotypes under different individual or combined stresses and/or the identification and testing of priming agents with potential to mitigate the adverse stress effects.

## Author Contributions

CM, LU, JH, LS, and NDD designed the experiments. CM and LU performed the experiments. JH performed the protocol for automated phenotyping, image processing, and image-based data analysis. TF developed the software routine for projected canopy height. MP and NDD performed the statistical analysis of the data, KP, SĆZ, and NDD performed the metabolite quantification. NDD and LS supervised the study and formulated the concept of the project. All authors discussed the results. CM, NDD, and LS wrote the manuscript.

## Conflict of Interest

The authors declare that the research was conducted in the absence of any commercial or financial relationships that could be construed as a potential conflict of interest.

## Abbreviations

(AAs), amino acids; (GABA), γ-aminobutyric acid; (APX), ascorbate peroxidase; (CAT), catalase; (POX), guaiacol peroxidase; (DAP), 1,3-diaminoporpane; (Ala), l-alanine; (Agm), agmatine; (Arg)¸ l-arginine; (Asn), l-asparagine; (Asp), l-aspartic acid; (Cit), l-citrulline; (Gln), l-glutamine; (Glu), l-glutamic acid; (Gly), l-glycine; (His), l-histidine; (Ile), l-ileucine; (Leu), l-leucine; (Met), l-methionine; (Phe), l-phenylalanine; (Pro), l-proline; (Ser), l-serine; (Trp), l-tryptophan; (Tyr), l-tyrosine; (PAs), polyamines; (Put), putrescine; (ROS), reactive oxygen species; (Spd), spermidine; (Spm), spermine; (Cad), cadaverine; (RGB), red, green, blue; (Tyra), tyramine; (Tryp), tryptamine.
