# Supplementary material for: A Novel Image-Based Screening Method to Study Water-Deficit Response and Recovery of Barley Populations Using Canopy Dynamics Phenotyping and Simple Metabolite Profiling
Source: Front Plant Sci. 2019 Oct 15;10:1252. doi: 10.3389/fpls.2019.01252 (PMC6804369; doi:10.3389/fpls.2019.01252)
Supplement: Supplementary file 1 [file DataSheet_1.pdf]

Supplementary Table S1. Scheme of the three performed experiments.

Experiment	Date	Growth conditions
1st	December 2017	Control
2nd	June 2018	
3rd	July 2018	Control *vs*. water deficit
