# Supplementary material for: A Novel Image-Based Screening Method to Study Water-Deficit Response and Recovery of Barley Populations Using Canopy Dynamics Phenotyping and Simple Metabolite Profiling
Source: Front Plant Sci. 2019 Oct 15;10:1252. doi: 10.3389/fpls.2019.01252 (PMC6804369; doi:10.3389/fpls.2019.01252)
Supplement: Supplementary file 3 [file DataSheet_3.pdf]

Supplementary Table S3. Statistical analysis of morphological and physiological traits in barley plants at the end of the water stress period and after rewatering using two-way ANOVA in R 3.5.1 Software.

		WATER STRESS					REWATERING				
		Df	Sum Sq	Mean Sq	F value	Pr(>F)	Df	Sum Sq	Mean Sq	F value	Pr(>F)
Biomass	TRAY	1	0.063	0.063	8.505	0.0069 **	1	0.003	0.003	0.142	0.712
	Treatment	1	8.333	8.333	1122.907	<2e-16 ***	1	3.547	3.547	194.408	2.27e-10 ***
	TRAY:Treatment	1	0.029	0.029	3.923	0.0575	1	0.002	0.002	0.088	0.771
	Residuals	28	0.208	0.007			16	0.292	0.018		
Leaf Length	TRAY	1	0.78	0.78	0.228	0.637	1	2.24	2.244	0.583	0.456
	Treatment	1	178.60	178.60	52.062	7.46e-08 ***	1	23.54	23.544	6.112	0.025 *
	TRAY:Treatment	1	0.60	0.60	0.176	0.678	1	0.00	0.005	0.001	0.973
	Residuals	28	96.06	3.43			16	61.64	3.852		
Leaf Width	TRAY	1	0.0013	0.0013	0.479	0.495	1	0.0005	0.00050	0.154	0.7001
	Treatment	1	0.9800	0.9800	375.248	<2e-16 ***	1	0.0125	0.01250	3.846	0.0675
	TRAY:Treatment	1	0.0003	0.0003	0.120	0.732	1	0.0125	0.01250	3.846	0.0675
	Residuals	28	0.0731	0.0026			16	0.0520	0.00325		
Ratio L/W	TRAY	1	7.5	7.5	0.555	0.463	1	7.78	7.778	1.148	0.300
	Treatment	1	769.0	769.0	57.022	3.17e-08 ***	1	5.22	5.225	0.771	0.393
	TRAY:Treatment	1	2.4	2.4	0.180	0.675	1	12.76	12.756	1.883	0.189
	Residuals	28	377.6	13.5			16	108.41	6.775		
Chl	TRAY	1	1.02	1.015	0.257	0.6161	1	4.05	4.050	1.400	0.254
	Treatment	1	29.45	29.453	7.459	0.0108 *	1	1.06	1.058	0.366	0.554
	TRAY:Treatment	1	0.20	0.195	0.049	0.8256	1	3.70	3.698	1.279	0.275
	Residuals	28	110.56	3.948			16	46.27	2.892		
RWC	TRAY	1	8	8	0.538	0.474	1	40.4	40.42	1.414	0.252
	Treatment	1	16318	16318	1146.045	2.55e-16 ***	1	1.5	1.46	0.051	0.824
	TRAY:Treatment	1	16	16	1.138	0.302	1	39.2	39.22	1.372	0.259
	Residuals	16	228	14			16	457.3	28.58		
CAT	TRAY	1	0.00239	0.00239	0.599	0.461063	1	0.01703	0.017028	1.433	0.266
	Treatment	1	0.11435	0.11435	28.646	0.000684 ***	1	0.02372	0.023716	1.996	0.195
	TRAY:Treatment	1	0.00007	0.00007	0.016	0.901389	1	0.00369	0.003695	0.311	0.592
	Residuals	8	0.03194	0.00399			8	0.09506	0.011882		
POX	TRAY	1	0.02	0.02	0.019	0.894008	1	0.0590	0.0590	0.596	0.4623
	Treatment	1	37.82	37.82	32.020	0.000477 ***	1	0.2248	0.2248	2.272	0.1701
	TRAY:Treatment	1	0.01	0.01	0.012	0.915079	1	0.5467	0.5467	5.525	0.0466 *
	Residuals	8	9.45	1.18			8	0.7916	0.0989		
APX	TRAY	1	0.009	0.009	0.009	0.9263	1	0.523	0.523	0.321	0.58648
	Treatment	1	4.080	4.080	4.074	0.0783	1	20.834	20.834	12.789	0.00723 **
	TRAY:Treatment	1	0.197	0.197	0.196	0.6695	1	0.012	0.012	0.007	0.93400
	Residuals	8	8.012	1.002			8	13.032	1.629		
