# Supplementary material for: A Novel Image-Based Screening Method to Study Water-Deficit Response and Recovery of Barley Populations Using Canopy Dynamics Phenotyping and Simple Metabolite Profiling
Source: Front Plant Sci. 2019 Oct 15;10:1252. doi: 10.3389/fpls.2019.01252 (PMC6804369; doi:10.3389/fpls.2019.01252)
Supplement: Supplementary file 4 [file DataSheet_4.pdf]

Supplementary Table S4. Statistical analysis of AAs and PAS (pmol mg^{-1} DW) in barley plants at the end of the water stress period and after rewatering using one-way ANOVA in R 3.5.1 Software.

	WATER STRESS	REWATERING
Ala	Df Sum Sq Mean Sq F value Pr(>F)<br>Treat 1 3.444 3.444 46.23 0.00244 **<br>Residuals 4 0.298 0.074	Df Sum Sq Mean Sq F value Pr(>F)<br>Treat 1 0.05929 0.05929 57.86 0.0016 **<br>Residuals 4 0.00410 0.00102
Arg	Df Sum Sq Mean Sq F value Pr(>F)<br>Treat 1 0.5545 0.5545 44.01 0.00268 **<br>Residuals 4 0.0504 0.0126	Df Sum Sq Mean Sq F value Pr(>F)<br>Treat 1 0.0001913 1.913e-04 2.557 0.185<br>Residuals 4 0.0002994 7.485e-05
Asn	Df Sum Sq Mean Sq F value Pr(>F)<br>Treat 1 18.289 18.289 58.88 0.00155 **<br>Residuals 4 1.242 0.311	Df Sum Sq Mean Sq F value Pr(>F)<br>Treat 1 0.1205 0.12052 193.1 0.000155 ***<br>Residuals 4 0.0025 0.00062
Asp	Df Sum Sq Mean Sq F value Pr(>F)<br>Treat 1 41.54 41.54 87.08 0.000734 ***<br>Residuals 4 1.91 0.48	Df Sum Sq Mean Sq F value Pr(>F)<br>Treat 1 1.1986 1.1986 33.68 0.00438 **<br>Residuals 4 0.1423 0.0356
Cit	Df Sum Sq Mean Sq F value Pr(>F)<br>Treat 1 5.102e-05 5.102e-05 28.65 0.00587 **<br>Residuals 4 7.120e-06 1.780e-06	Df Sum Sq Mean Sq F value Pr(>F)<br>Treat 1 0.0003207 0.0003207 65.41 0.00127 **<br>Residuals 4 0.0000196 0.0000049
GABA	Df Sum Sq Mean Sq F value Pr(>F)<br>Treat 1 0.9506 0.9506 25.35 0.00731 **<br>Residuals 4 0.1500 0.0375	Df Sum Sq Mean Sq F value Pr(>F)<br>Treat 1 0.02496 0.024964 6.389 0.0648<br>Residuals 4 0.01563 0.003907
Gln	Df Sum Sq Mean Sq F value Pr(>F)<br>Treat 1 14.696 14.696 712.8 1.17e-05 ***<br>Residuals 4 0.082 0.021	Df Sum Sq Mean Sq F value Pr(>F)<br>Treat 1 2.1068 2.1068 187.9 0.000164 ***<br>Residuals 4 0.0449 0.0112
Glu	Df Sum Sq Mean Sq F value Pr(>F)<br>Treat 1 33.67 33.67 64.78 0.00129 **<br>Residuals 4 2.08 0.52	Df Sum Sq Mean Sq F value Pr(>F)<br>Treat 1 0.9953 0.9953 16.94 0.0147 *<br>Residuals 4 0.2350 0.0588
Gly	Df Sum Sq Mean Sq F value Pr(>F)<br>Treat 1 0.005175 0.005175 13.88 0.0204 *<br>Residuals 4 0.001492 0.000373	Df Sum Sq Mean Sq F value Pr(>F)<br>Treat 1 0.005175 0.005175 13.88 0.0204 *<br>Residuals 4 0.001492 0.000373
His	Df Sum Sq Mean Sq F value Pr(>F)<br>Treat 1 1.343 1.3431 358.5 4.58e-05 ***<br>Residuals 4 0.015 0.0037	Df Sum Sq Mean Sq F value Pr(>F)<br>Treat 1 0.003950 0.003950 114 0.000436 ***<br>Residuals 4 0.000139 0.000035
Ile	Df Sum Sq Mean Sq F value Pr(>F)<br>Treat 1 2.1271 2.1271 333.5 5.29e-05 ***<br>Residuals 4 0.0255 0.0064	Df Sum Sq Mean Sq F value Pr(>F)<br>Treat 1 0.0008674 0.0008674 14.67 0.0186 *<br>Residuals 4 0.0002366 0.0000591
Leu	Df Sum Sq Mean Sq F value Pr(>F)<br>Treat 1 1.0025 1.0025 369.7 4.31e-05 ***<br>Residuals 4 0.0108 0.0027	Df Sum Sq Mean Sq F value Pr(>F)<br>Treat 1 0.0003770 3.77e-04 14.24 0.0195 *<br>Residuals 4 0.0001059 2.65e-05
Met	Df Sum Sq Mean Sq F value Pr(>F)<br>Treat 1 0.015151 0.015151 705.5 1.19e-05 ***<br>Residuals 4 0.000086 0.000021	Df Sum Sq Mean Sq F value Pr(>F)<br>Treat 1 3.074e-05 3.074e-05 237.7 0.000103 ***<br>Residuals 4 5.170e-07 1.290e-07
Phe	Df Sum Sq Mean Sq F value Pr(>F)<br>Treat 1 0.3306 0.3306 83805 8.54e-10 ***<br>Residuals 4 0.0000 0.0000	Df Sum Sq Mean Sq F value Pr(>F)<br>Treat 1 0.0011638 0.0011638 28.12 0.00608 **<br>Residuals 4 0.0001656 0.0000414

OH-Pro	Df Sum Sq Mean Sq F value Pr(>F)<br>Treat 1 0.0007348 0.0007348 166.4 0.000208 ***<br>Residuals 4 0.0000177 0.0000044	Df Sum Sq Mean Sq F value Pr(>F)<br>Treat 1 9.210e-07 9.21e-07 26.39 0.00681 **<br>Residuals 4 1.396e-07 3.49e-08
Pro	Df Sum Sq Mean Sq F value Pr(>F)<br>Treat 1 11410 11410 289.4 7e-05 ***<br>Residuals 4 158 39	Df Sum Sq Mean Sq F value Pr(>F)<br>Treat 1 11.947 11.947 8.653 0.0423 *<br>Residuals 4 5.522 1.381
Ser	Df Sum Sq Mean Sq F value Pr(>F)<br>Treat 1 15.890 15.890 234.6 0.000106 ***<br>Residuals 4 0.271 0.068	Df Sum Sq Mean Sq F value Pr(>F)<br>Treat 1 0.12556 0.1256 26.69 0.00667 **<br>Residuals 4 0.01882 0.0047
Trp	Df Sum Sq Mean Sq F value Pr(>F)<br>Treat 1 1.3558 1.3558 12212 4.02e-08 ***<br>Residuals 4 0.0004 0.0001	Df Sum Sq Mean Sq F value Pr(>F)<br>Treat 1 0.013065 0.013065 16.39 0.0155 *<br>Residuals 4 0.003188 0.000797
Tyr	Df Sum Sq Mean Sq F value Pr(>F)<br>Treat 1 0.4327 0.4327 30796 6.33e-09 ***<br>Residuals 4 0.0001 0.00003	Df Sum Sq Mean Sq F value Pr(>F)<br>Treat 1 0.0004264 0.0004264 25.59 0.00718 **<br>Residuals 4 0.0000666 0.0000167
AAs	Df Sum Sq Mean Sq F value Pr(>F)<br>Treat 1 19930 19930 227.9 0.000112 ***<br>Residuals 4 350 87	Df Sum Sq Mean Sq F value Pr(>F)<br>Treat 1 72.15 72.15 26.54 0.00674 **<br>Residuals 4 10.87 2.72
Cad	Df Sum Sq Mean Sq F value Pr(>F)<br>Treat 1 115.79 115.79 262.2 8.51e-05 ***<br>Residuals 4 1.77 0.44	
DAP	Df Sum Sq Mean Sq F value Pr(>F)<br>Treat 1 178.03 178.03 11682 4.39e-08 ***<br>Residuals 4 0.06 0.02	Df Sum Sq Mean Sq F value Pr(>F)<br>Treat 1 8.214 8.214 147.3 0.000264 ***<br>Residuals 4 0.223 0.056
Put	Df Sum Sq Mean Sq F value Pr(>F)<br>Treat 1 328.2 328.2 2220 1.21e-06 ***<br>Residuals 4 0.6 0.1	Df Sum Sq Mean Sq F value Pr(>F)<br>Treat 1 3.988 3.988 4.684 0.0964 .<br>Residuals 4 3.406 0.851
Spd	Df Sum Sq Mean Sq F value Pr(>F)<br>Treat 1 1.492 1.492 0.956 0.384<br>Residuals 4 6.244 1.561	Df Sum Sq Mean Sq F value Pr(>F)<br>Treat 1 160.45 160.45 9.114 0.0392 *<br>Residuals 4 70.42 17. 61
Spm	Df Sum Sq Mean Sq F value Pr(>F)<br>Treat 1 475.4 475.4 22.8 0.00881 **<br>Residuals 4 83.4 20.9	Df Sum Sq Mean Sq F value Pr(>F)<br>Treat 1 9.838 9.838 3.868 0.121<br>Residuals 4 10.172 2.543
Tyra	Df Sum Sq Mean Sq F value Pr(>F)<br>Treat 1 11955 11955 135.8 0.00031 ***<br>Residuals 4 352 88	Df Sum Sq Mean Sq F value Pr(>F)<br>Treat 1 179.92 179.92 16.99 0.0146 *<br>Residuals 4 42.36 10.59
PAs	Df Sum Sq Mean Sq F value Pr(>F)<br>Treat 1 26044 26044 134.6 0.000315 ***<br>Residuals 4 774 193	Df Sum Sq Mean Sq F value Pr(>F)<br>Treat 1 1161.4 1161.4 73.11 0.00103 **<br>Residuals 4 63.5 15.9
