# Supplementary figures and images for: A Novel Image-Based Screening Method to Study Water-Deficit Response and Recovery of Barley Populations Using Canopy Dynamics Phenotyping and Simple Metabolite Profiling
Source: Front Plant Sci. 2019 Oct 15;10:1252. doi: 10.3389/fpls.2019.01252 (PMC6804369; doi:10.3389/fpls.2019.01252)

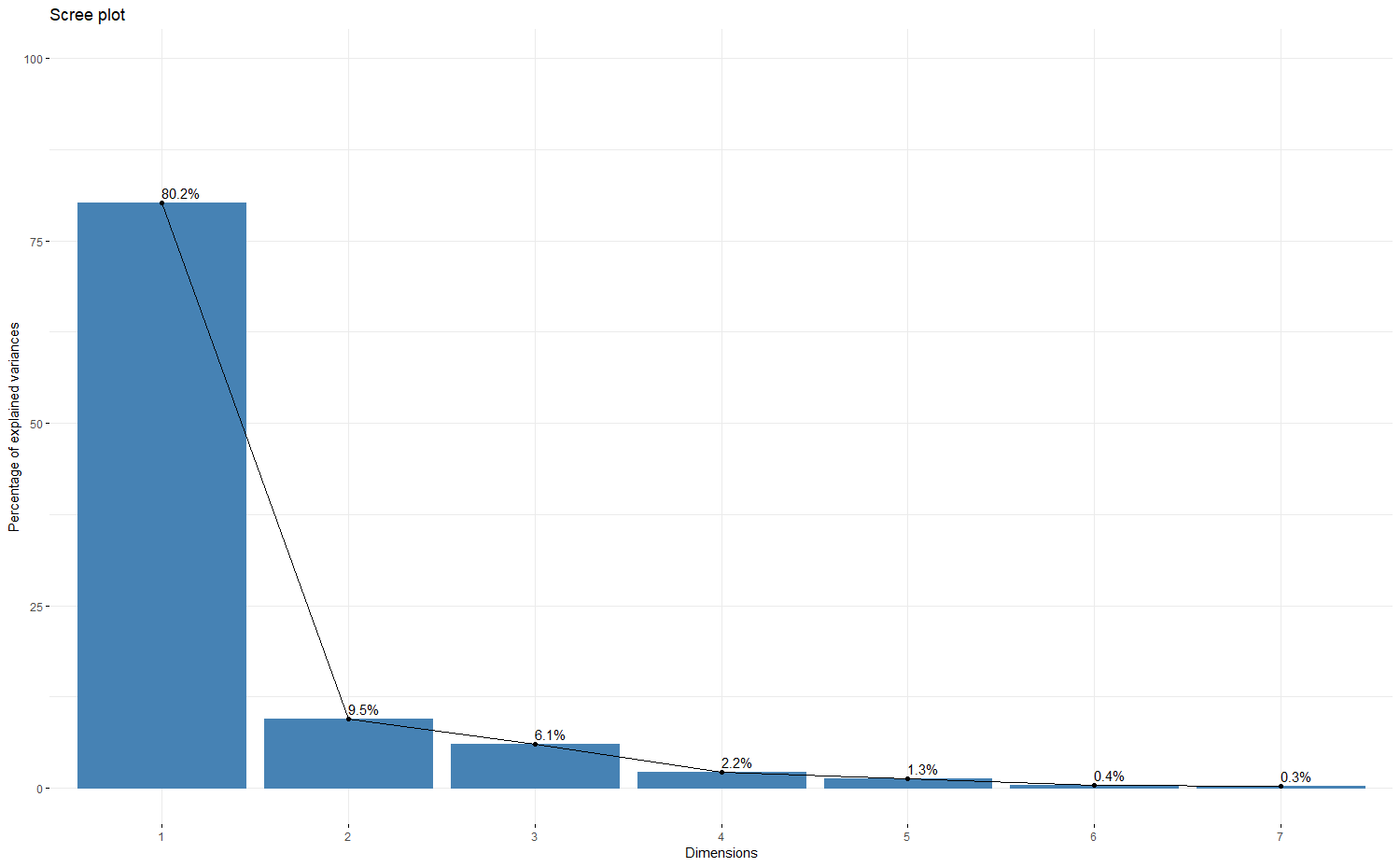

Supplement: Supplementary file 6 [file Image_2.tiff]

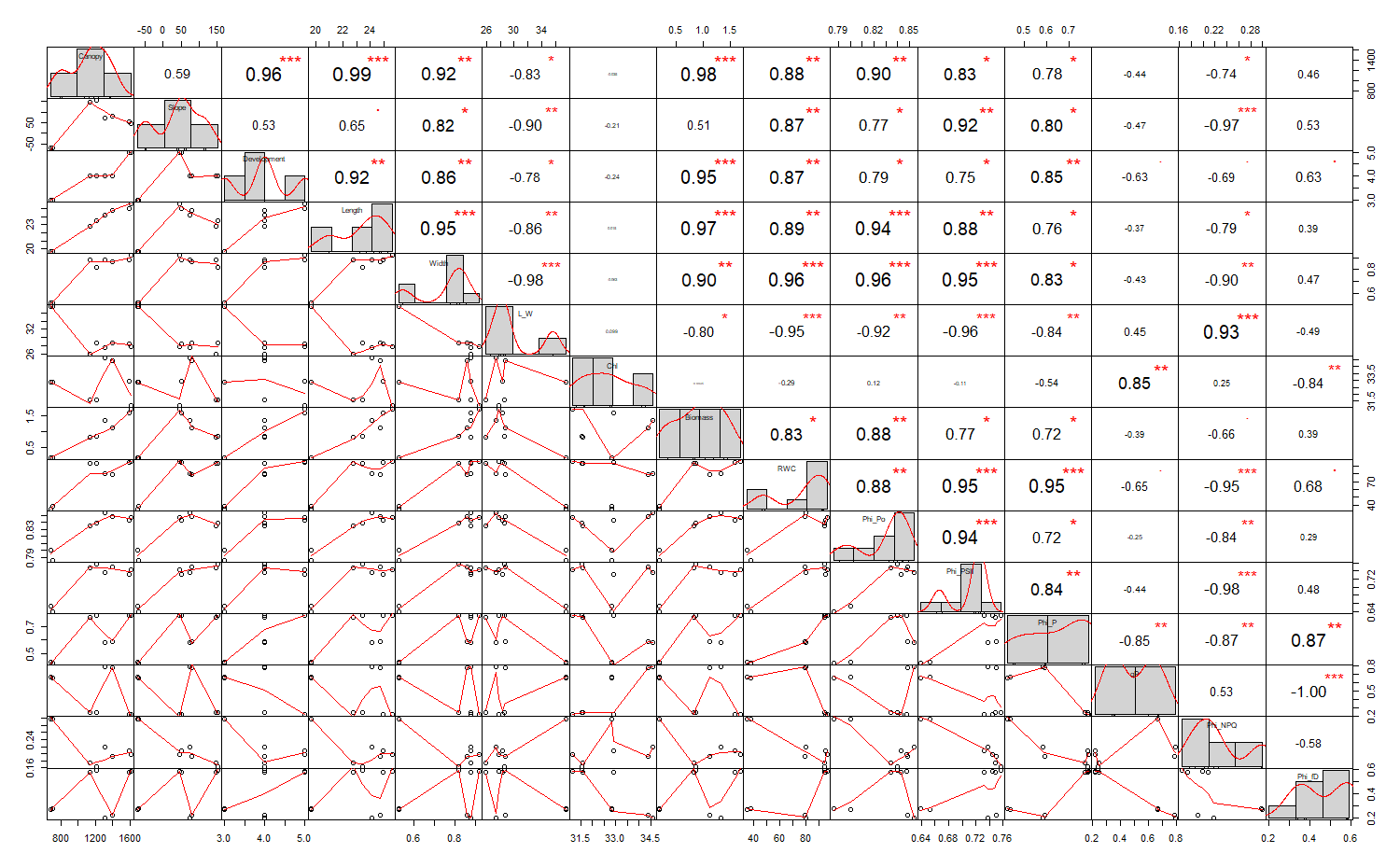

Supplement: Supplementary file 7 [file Image_3.tiff]

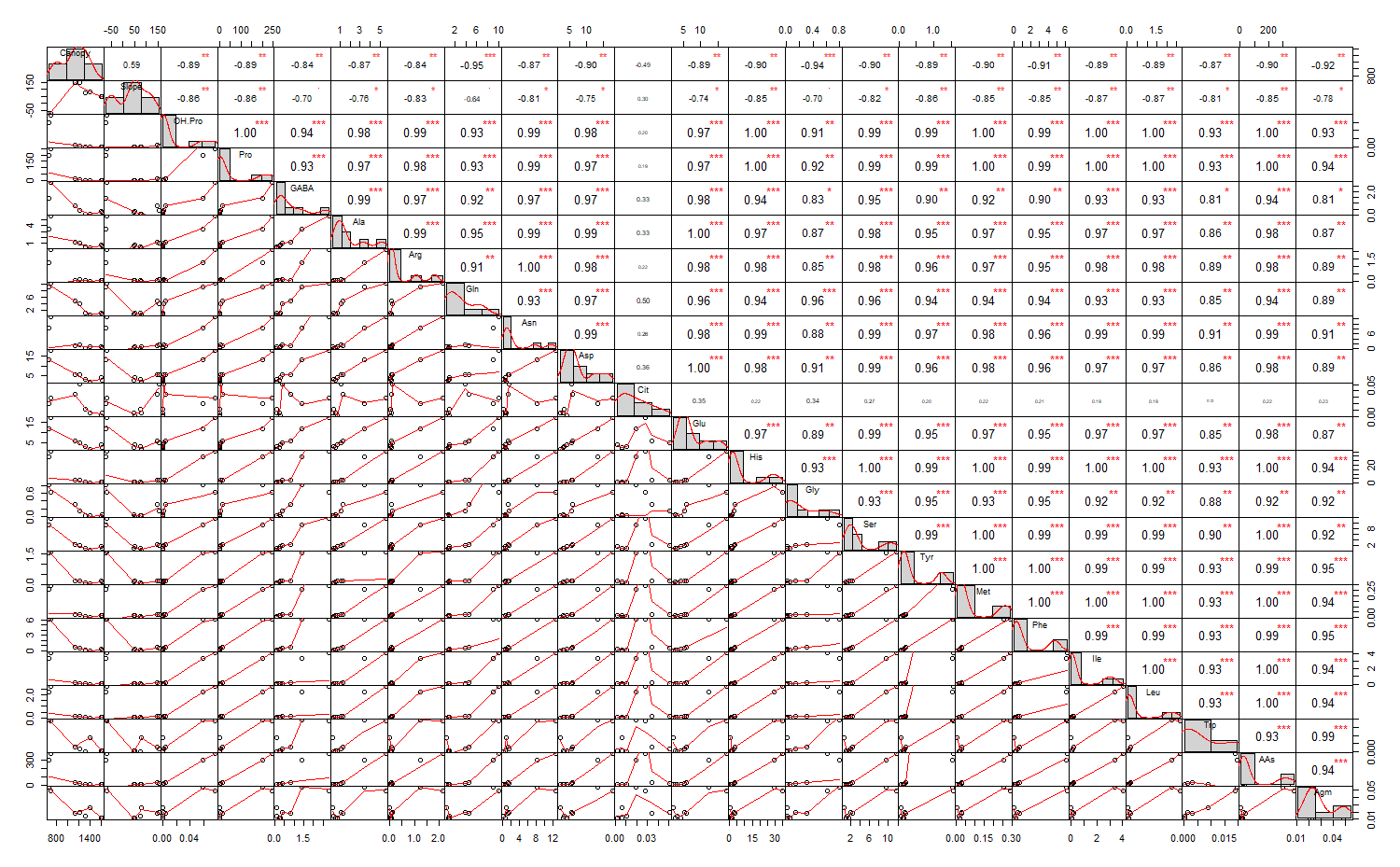

Supplement: Supplementary file 8 [file Image_4.tiff]

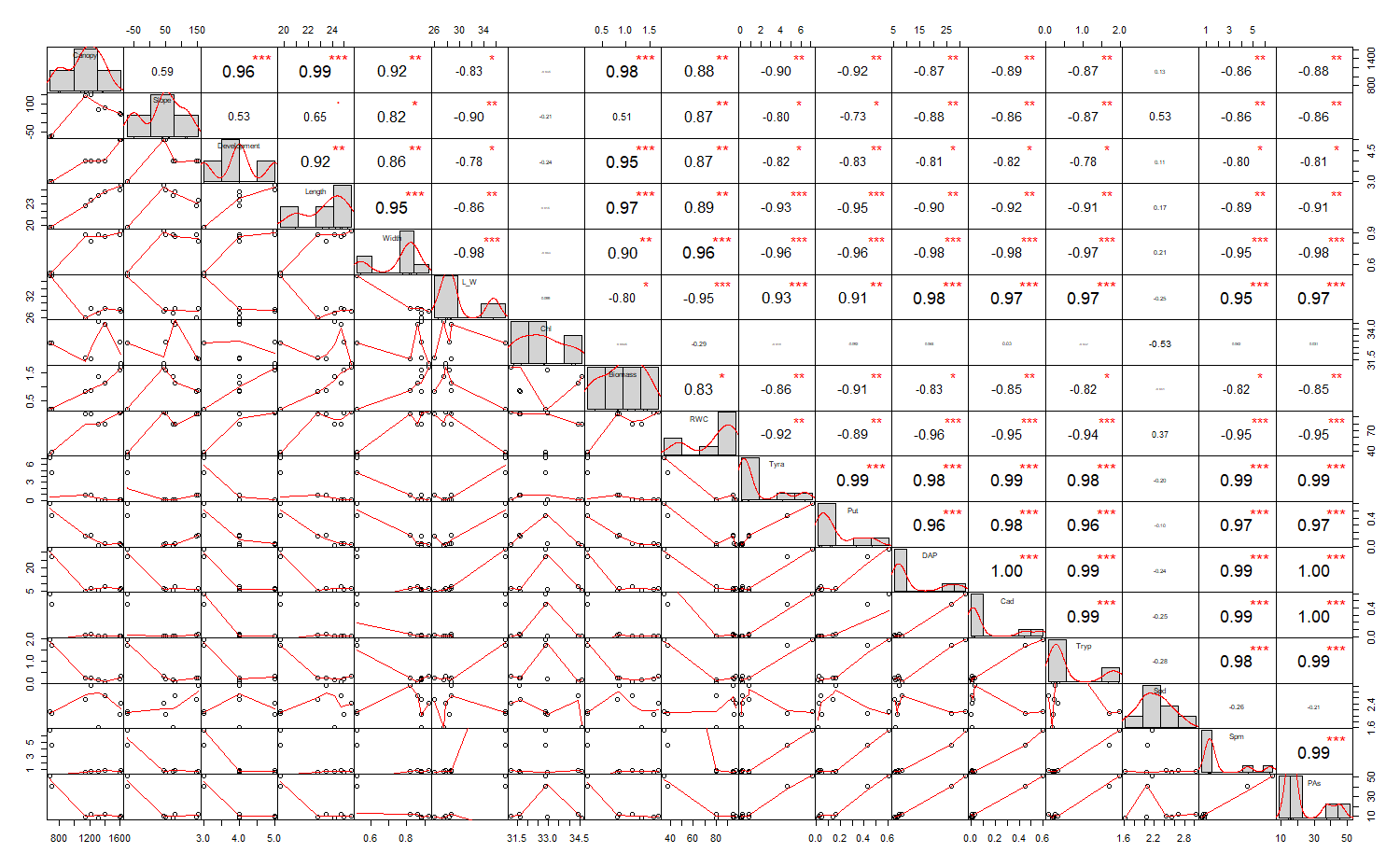

Supplement: Supplementary file 9 [file Image_5.tiff]

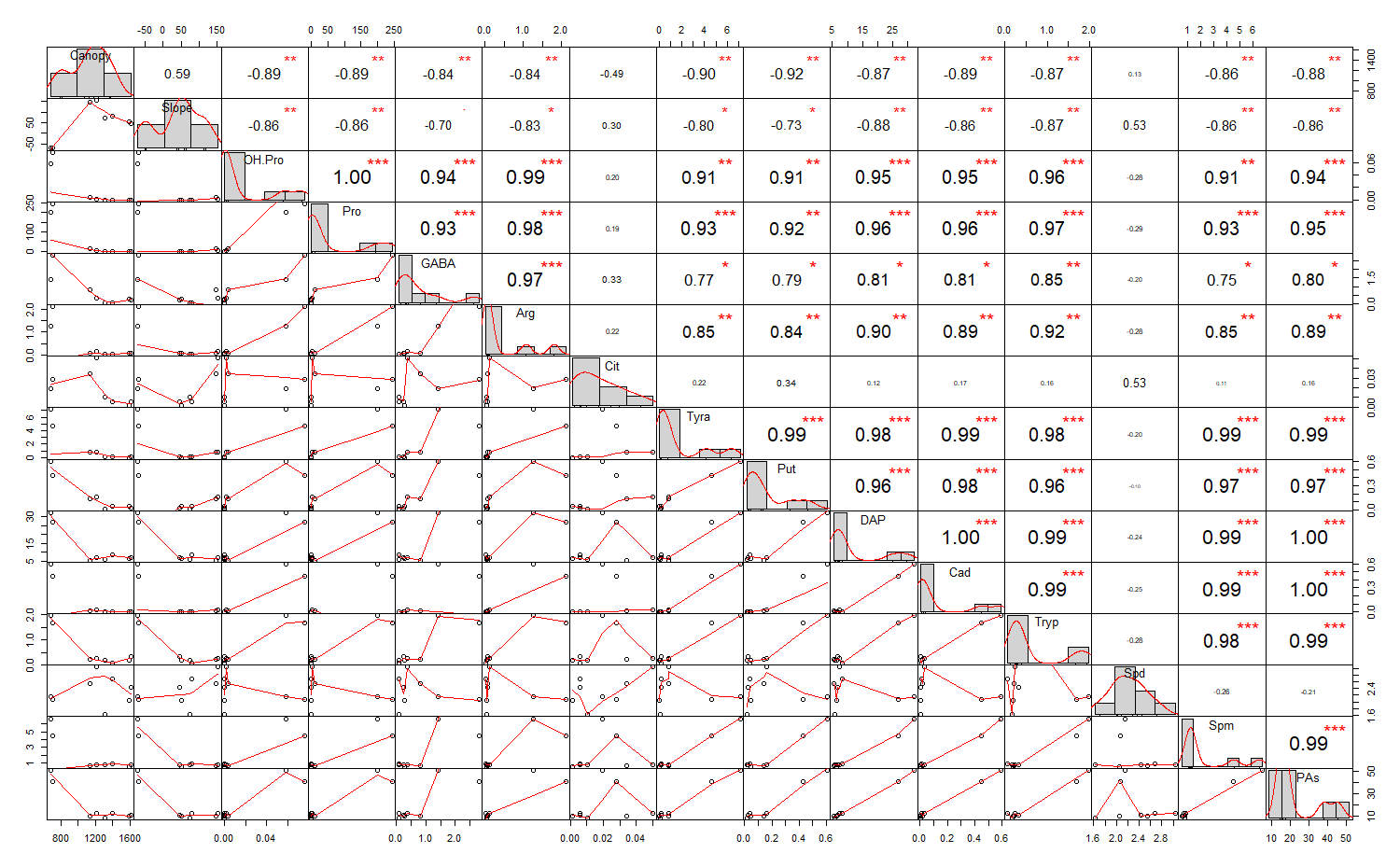

Supplement: Supplementary file 10 [file Image_6.tiff]

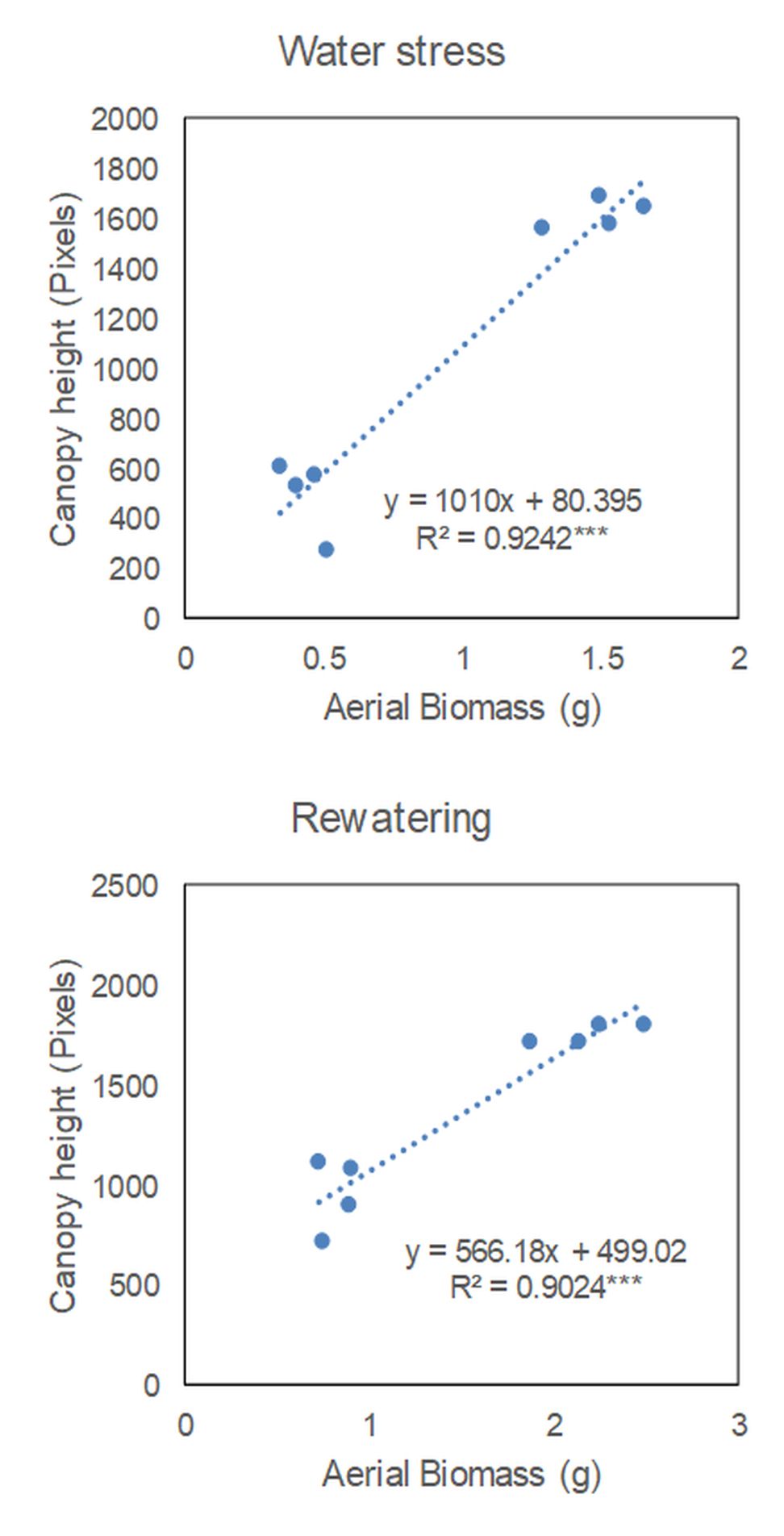

Supplement: Supplementary file 11 [file Image_7.jpeg]

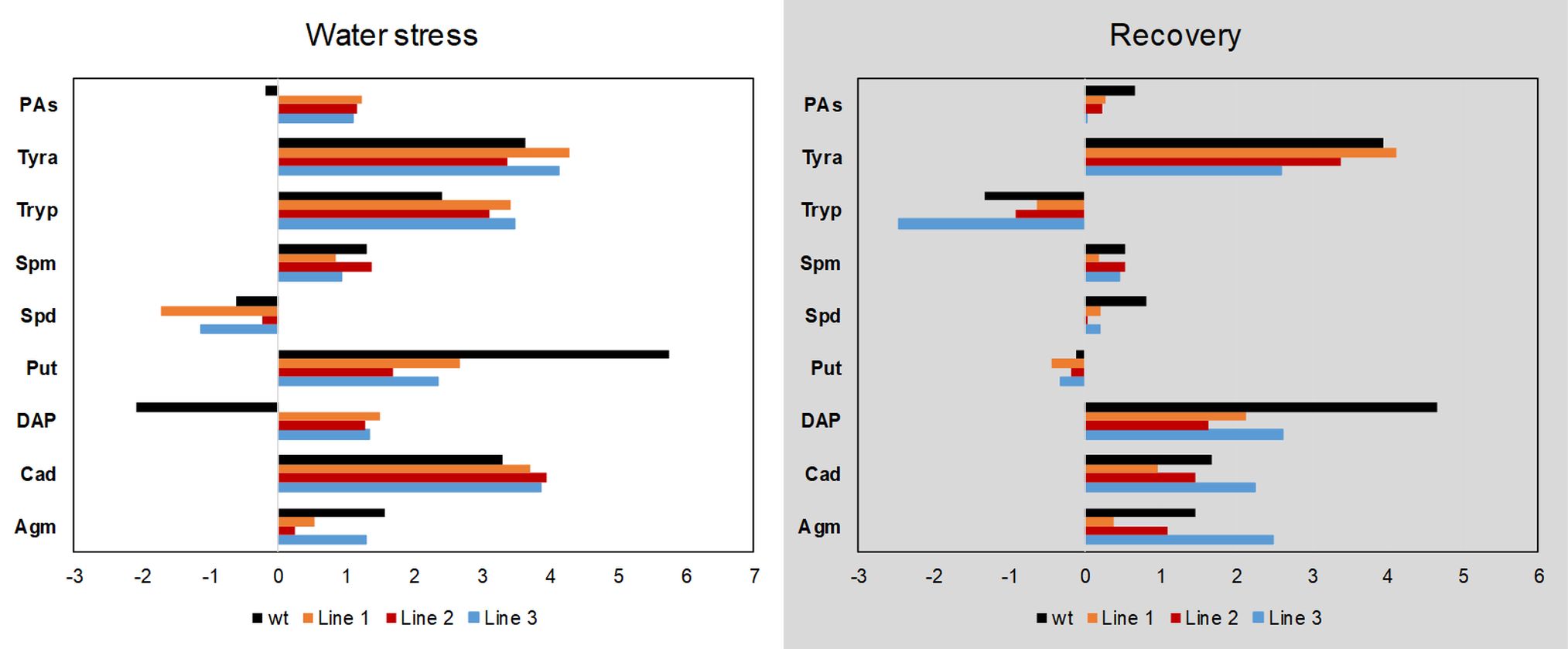

Supplement: Supplementary file 12 [file Image_8.jpeg]

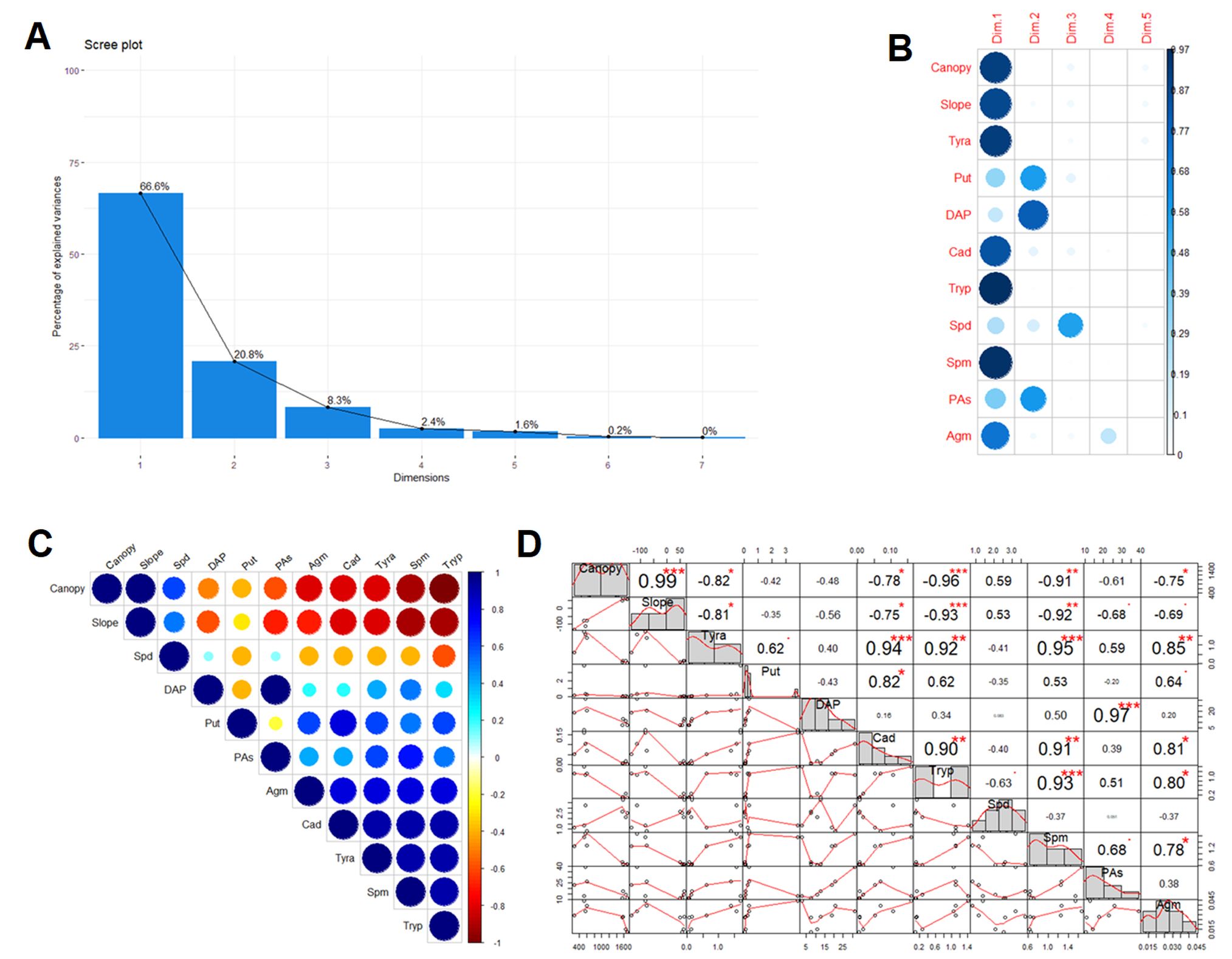

Supplement: Supplementary file 13 [file Image_9.jpeg]

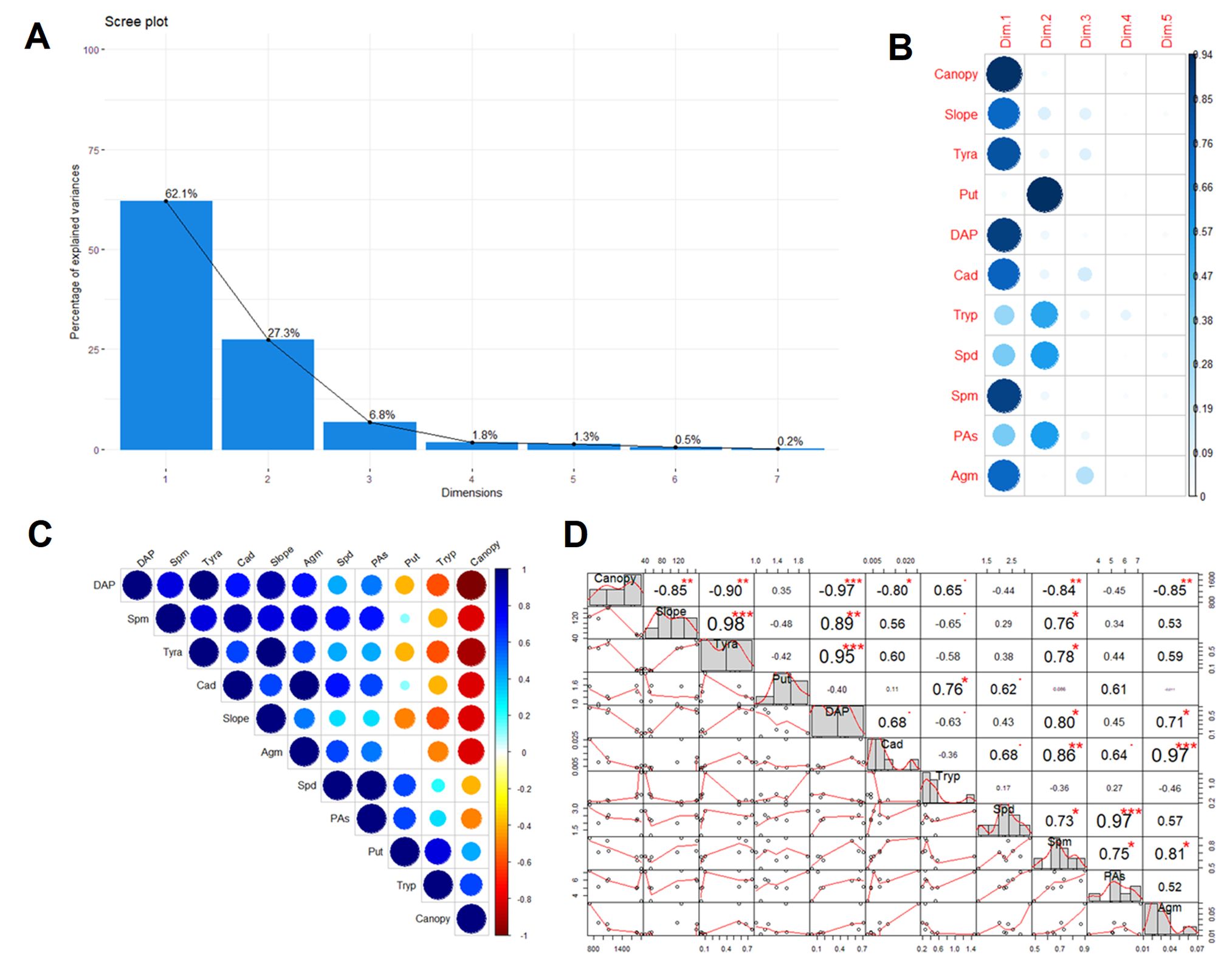

Supplement: Supplementary file 14 [file Image_10.jpeg]
