# Supplementary material for: A Novel Image-Based Screening Method to Study Water-Deficit Response and Recovery of Barley Populations Using Canopy Dynamics Phenotyping and Simple Metabolite Profiling
Source: Front Plant Sci. 2019 Oct 15;10:1252. doi: 10.3389/fpls.2019.01252 (PMC6804369; doi:10.3389/fpls.2019.01252)
Supplement: Supplementary file 15 [file Presentation_1.pdf]

Supplementary Figure S1| Analysis of reproducibility of canopy height estimation in barley (*Hordeum vulgare*) seedling under water deficit conditions within experimental replicates. Changes in canopy height (in pixels) of stressed (D, discontinuous lines) or non-stressed (W, continuous lines) barley seedlings (n=50) from five independent trays grown for 12 days (with the endpoint at day 15)

Supplementary Figure S2| Scree plot representing the percentage of explained variance of the model of each PC analysis (Dim) performed in R3.5.1

Supplementary Figure S3| Scatter plots of the correlation between canopy height, slope, morphometric parameters, RWC (%), chl and fluoresce related traits undertaken in R3.5.1.

Supplementary Figure S4| Scatter plots of the correlation between canopy height, slope and free AAs undertaken in R3.5.1.

Supplementary Figure S5| Scatter plots of the correlation between canopy height, slope, CAT, POX and APX and free PAS undertaken in R3.5.1.

Supplementary Figure S6| Scatter plots of the correlation between the activity of canopy height, slope, morphometric parameters, RWC (%), Pro, OH-Pro, GABA, Arg, Cit and free PAS undertaken in R3.5.1.

Supplementary Figure S7| Correlation between biomass and canopy height of three different transgenic barley lines and wt at the end of the water stress period and after rewatering.

Supplementary Figure S8| Metabolic profiles of three transgenic lines and wt of barley under water deficit and after subsequent rewatering. Fold changes (presented as $\log_2$ ratio) in the content (pmol mg^{-1} DW) of free polyamines (PAs) between stressed (D) and non-stressed (W) barley seedlings (four independent pools cotaining 5 plants from each variant).

Supplementary Figure S9| Scree plot representing the percentage of explained variance of the model of each PC analysis (Dim) (a) contribution of the loadings to each PCA (Dim) (b) and correlation matrices (c and d) with the significance of 11 variables including 2 traits and 9 free PAs obtained from three different transgenic barley lines and wt at the end of the water stress period, all performed in R3.5.1.

Supplementary Figure S10| Scree plot representing the percentage of explained variance of the model of each PC analysis (Dim) (a) contribution of the loadings to each PCA (Dim) (b) and correlation matrices (c and d) with the significance of 11 variables including 2 traits and 9 free

PAs obtained from three different transgenic barley lines and wt after rewatering, all performed in R3.5.1.

Supplementary Video S1| Movie file of the projected canopy height in barley seedlings growing under water stress conditions with subsequent rewatering.
