# Supplementary material for: A Novel Image-Based Screening Method to Study Water-Deficit Response and Recovery of Barley Populations Using Canopy Dynamics Phenotyping and Simple Metabolite Profiling
Source: Front Plant Sci. 2019 Oct 15;10:1252. doi: 10.3389/fpls.2019.01252 (PMC6804369; doi:10.3389/fpls.2019.01252)
Supplement: Supplementary file 2 [file DataSheet_2.pdf]

Supplementary Table S2. Dynamic of canopy height in barley seedling under drought conditions and rewatering. Fold changes (log_2) of the canopy height of stressed seedlings under water stress conditions for 13 days (Day 16) and subsequent rewatering for 4 days (from day 16 to day 19) compared to their respective controls monitored in three independent biological replicates. Asterisks show significance between treated and non-treated plants according to ANOVA (n=50).

TRAY	Day 1	Day 2	Day 3	Day 4	Day 5	Day 6	Day 7	Day 8	Day 9	Day 10	Day 11	Day 12	Day 13	Day 14	Day 15	Day 16	Day 17	Day 18	Day 19
Tray 1	-0.12	-0.28	-0.20	-0.13	-0.18	-0.11	-0.13	-0.06	-0.05	-0.07	-0.16	-0.36	-0.72	-0.89	-0.96	-1.00	-0.66	-0.58	-0.40
Tray 2	-0.20	-0.05	-0.08	-0.12	-0.12	-0.04	-0.05	0.00	-0.14	-0.05	-0.21	-0.46	-0.72	-0.81	-0.98	-1.00	-0.78	-0.64	-0.49
Tray 3	-0.08	-0.04	-0.06	0.02	0.02	0.04	-0.03	0.06	-0.06	-0.06	-0.27	-0.43	-0.75	-0.92	-1.03	-1.11	-0.87	-0.73	-0.65
**Average**	**-0.13**	**-0.12**	**-0.11**	**-0.07**	**-0.09**	**-0.04**	**-0.07**	**0.00**	**-0.08**	**-0.06**	**-0.21**	**-0.41**	**-0.73**	**-0.87**	**-0.99**	**-1.04**	**-0.77**	**-0.65**	**-0.51**
P-value	ns	ns	ns	ns	ns	ns	ns	ns	ns	ns	*	*	*	*	*	*	*	*	*
